# Active Learning-Guided Optimization of Moisture Swing Adsorption for Direct Air Capture

**DOI:** 10.1021/acs.est.6c07244

**Published:** 2026-09-26

**Authors:** Peiling Yu, Kai Zhang, Xiaoyang Shi, Zihan Zheng, Huichun Zhang

**Affiliations:** † Department of Civil and Environmental Engineering, 2546 Case Western Reserve University, Cleveland, Ohio 44106, United States; ‡ Department of Earth and Environmental Engineering, 5798 Columbia University, New York, New York 10027, United States

**Keywords:** Active learning, data-driven, design of experiments, direct air capture, moisture swing adsorption, optimization

## Abstract

Moisture swing adsorption (MSA) offers an energy-efficient route for direct air capture of CO_2_ by exploiting humidity-driven sorbent regeneration, yet its performance remains difficult to predict due to the coupled effects of sorbent chemistry, counterion properties, and operating conditions. Here, we present a data-efficient experimental and modeling strategy that systematically maps these interactions across a high-dimensional design space. Using active learning to target regions of maximal uncertainty, we expand experimental coverage from 19 to 77 conditions and, together with curated literature data, assemble a unified dataset of 290 data points (201 distinct experimental conditions) spanning 20 commercial ion-exchange resins. Interpretable machine-learning models trained on this dataset predict CO_2_ capture capacity (*R* ^2^ = 0.702, RMSE = 0.164) and identify functional groups, anion basicity, humidity, and CO_2_ concentration as dominant performance determinants. Guided by these insights, we experimentally validate model-selected resin–anion combinations for two practical scenarios—maximum CO_2_ capture capacity and moisture-swing efficiency. This work converts fragmented experimental observations into a predictive structure–property framework and demonstrates a generalizable approach for understanding and optimizing complex adsorption systems under data and experimental constraints.

## Graphical abstract

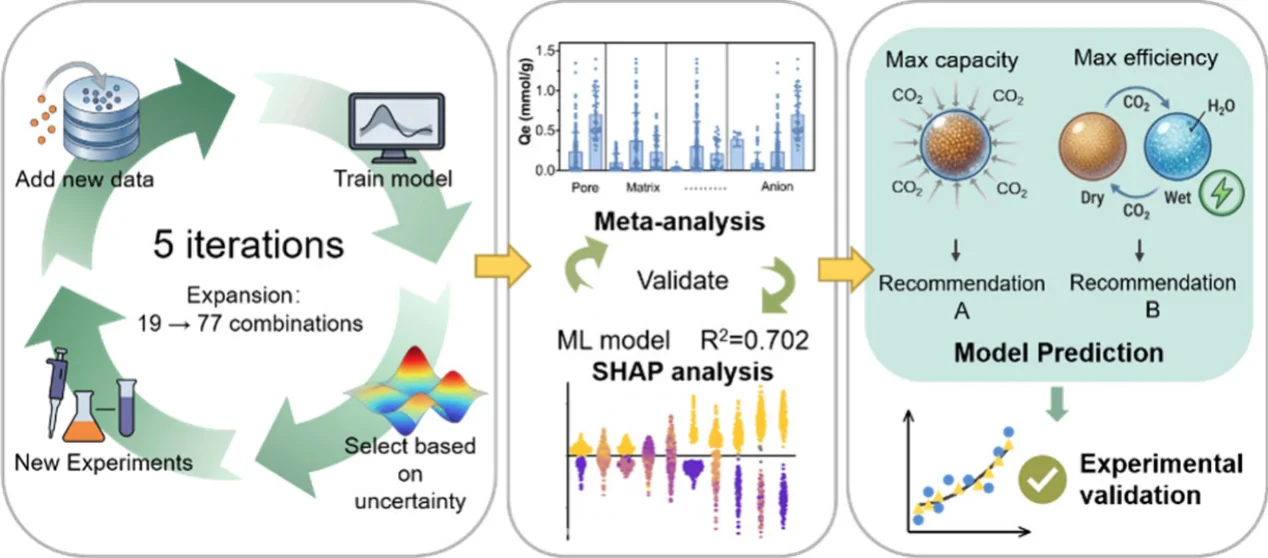

## 1 Introduction

The continuous accumulation of anthropogenic atmospheric carbon dioxide (CO_2_) is one of the primary contributors to climate change and the increasing frequency of extreme weather events.^1–3^ The Intergovernmental Panel on Climate Change (IPCC) highlights that simply reducing emissions is likely insufficient; deploying negative emission technologies is critical to balance the global carbon budget.^3^ The concept of directly capturing CO_2_ from ambient air was pioneered by Lackner et al.,^4^ and direct air capture (DAC) has since developed into a promising approach for extracting ultra-dilute CO_2_ (∼400 ppm) to remediate decentralized emissions.^5^ Achieving effective atmospheric CO_2_ removal requires specially designed adsorbents, including high-porosity physical sorbents (such as zeolites, activated carbon, alumina, and metal-organic frameworks)^6,7^ and chemical sorbents (e.g., amine-functionalized materials).^8^ While strong CO_2_ binding necessitates energy-intensive regeneration, with reported thermal energy demands of approximately 231 kJ/mol for liquid alkali hydroxides^9^ and 176–519 kJ/mol for solid sorbents,^10^ moisture-swing resins were estimated to require only about 50 kJ/mol for vacuum evacuation, CO_2_ compression, and water-vapor compression, assuming humidification itself requires no net external heat.^11^ This low regeneration energy positions them as a promising candidate for energy-efficient carbon capture.^12^

Moisture-swing sorbents are modulated by ambient humidity, where ammonium cations are covalently tethered to the material matrix as functional groups, and anions serve as counterions to maintain charge neutrality. These sorbents capture CO_2_ under dry conditions and release it upon exposure to humidity.^13,14^ Moisture swing adsorption (MSA) system performance is influenced by the physicochemical properties of the matrix, functional groups, and counterions. Regarding the matrix, pore architecture directly dictates adsorption kinetics and capacity.^15^ For instance, uniform cylindrical pores enhance functional group efficiency and capacity,^16^ while hierarchical micro-mesoporous structures accelerate adsorption kinetics by approximately 4-fold.^17^ Furthermore, pore size modulates the local microenvironment via the capillary condensation effect: at 20°C and 21% ambient relative humidity (RH), the local humidity within <10 nm pores can exceed 30%, activating the MSA mechanism under macroscopically dry conditions.^16^ Surface wettability is also critical: incorporation of hydrophobic polyvinylidene fluoride reduces humidity sensitivity and raises the equilibrium CO_2_ uptake from ∼0.56 to ∼0.90 mmol/g (at 20 °C, 84.7% RH, 400 ppm of CO_2_).^18^ The chemical nature of the functional groups and ions exerts an equally decisive influence. First, the functional group type directly impacts capacity: macroporous strong-base resins exhibit an adsorption capacity of ∼0.76 mmol/g, approximately 7 times that of weak-base counterparts.^15^ Building on this, increasing functional group density further enhances performance; a modified diamine-based bis-quaternary ammonium resin, with quaternary content raised to 68–81%, achieved a humidity-swing DAC capacity of 3.41 mmol/g.^19^ Moreover, the identity of the mobile counter-anion significantly affects capture capacity.^20^ For example, the resins loaded with phosphate outperform carbonate systems by 80% in swing capacity.^21^ The valency and basicity of these anions also modulate the thermodynamic behavior of the MSA process.^22–25^ Besides, environmental conditions also influence MSA-CO_2_ adsorption performance. For instance, introducing temperature variations into the MSA cycle can enhance adsorbent efficiency by 30%.^26^

Because matrix properties, functional-group chemistry, counterion characteristics, and environmental conditions jointly determine performance rather than acting independently, MSA constitutes a high-dimensional and strongly coupled system. Prior studies have provided valuable mechanistic insights and parameter-specific analyses; however, such approaches cannot readily resolve the interactions among coupled variables, identify which factor combinations dominate performance, or predict overall system behavior; fully integrating these factors into a system-level understanding remains challenging. Developing a unified structure-property framework and system-level predictive models would enable more rational materials design and more reliable guidance for optimizing MSA systems, but doing so requires an approach capable of learning multi-factor relationships from limited data.

Realizing this vision, however, fundamentally depends on the availability of robust and well-curated data. In data-driven adsorption prediction, the scarcity and fragmentation of high-quality datasets remain primary bottlenecks for constructing reliable machine learning (ML) models.^27^ Unlike conventional ML, which learns passively from a fixed, predetermined dataset, active learning (AL) is an iterative approach in which the model actively determines which data to acquire next, so that the dataset is built adaptively rather than fixed in advance. Moreover, exhaustive exploration of the full parameter space is resource intensive. AL offers a promising strategy by prioritizing high-information experiments to maximize knowledge gain per trial, thereby enhancing model performance while reducing experimental cost. In materials science, AL has been successfully deployed for the design and optimization of adsorbents and catalysts. For instance, studies have utilized AL to optimize synthesis conditions for activated carbon,^28^ eliminate redundant experiments,^29^ and identify key structure-property features in catalysis.^30^ More recently, AL-particle swarm optimization (PSO) frameworks have doubled CO_2_ uptake by targeting a single structural descriptor in porous carbon synthesis.^31^ Related closed-loop frameworks pair an iteratively updated model with optimization algorithms to tune methanol synthesis process parameters under data feedback.^32^ However, many AL and Bayesian optimization studies in materials discovery are formulated as targeted optimization problems, in which acquisition functions balance exploitation of high-performing predicted regions with exploration of uncertain regions to locate property extrema.^33,34^ While such strategies are effective for accelerating the discovery of optimal candidates, an acquisition objective dominated by optimum seeking can preferentially allocate measurements near promising or locally optimal regions and leave other parts of the design space less characterized.^34^ This limitation is particularly important for MSA, where adsorption performance emerges from coupled effects among multiple parameters. In this context, identifying the best-performing condition alone does not necessarily reveal how these factors jointly shape adsorption behavior across the broader parameter space. Therefore, MSA calls for an exploration-oriented AL strategy that goes beyond single-objective optimum seeking by sampling underrepresented regions, thereby enabling reconstruction of the multidimensional structure-property landscape and integration of new measurements with fragmented literature data.

To overcome the limitations of existing fragmented MSA datasets, this study adopts an exploration-oriented acquisition objective that strategically selects the most informative data points. We aim to efficiently reconstruct the entire response surface with minimal experimental effort, transforming fragmented data into a holistic understanding. In doing so, this work combines three elements: the reformulation of AL toward response-surface reconstruction, the integration of experimental and literature data, and the interpretation of coupled adsorption factors through SHAP analysis (Figure 1). The framework proceeds as follows: First, an AL strategy was employed to guide the design of experiments, ensuring efficient coverage of the multidimensional variable space with a limited number of experiments. Subsequently, these experimental data were integrated with literature data for meta-analysis, systematically evaluating the contributions of resin properties, ion characteristics, and environmental conditions to CO_2_ uptake. Finally, we developed, interpreted, and validated ML models to (1) predict anion exchange resin (AER)-MSA system performance, (2) quantify feature importance and interactions, and (3) guide optimal material design and process optimization.

**Figure 1:**
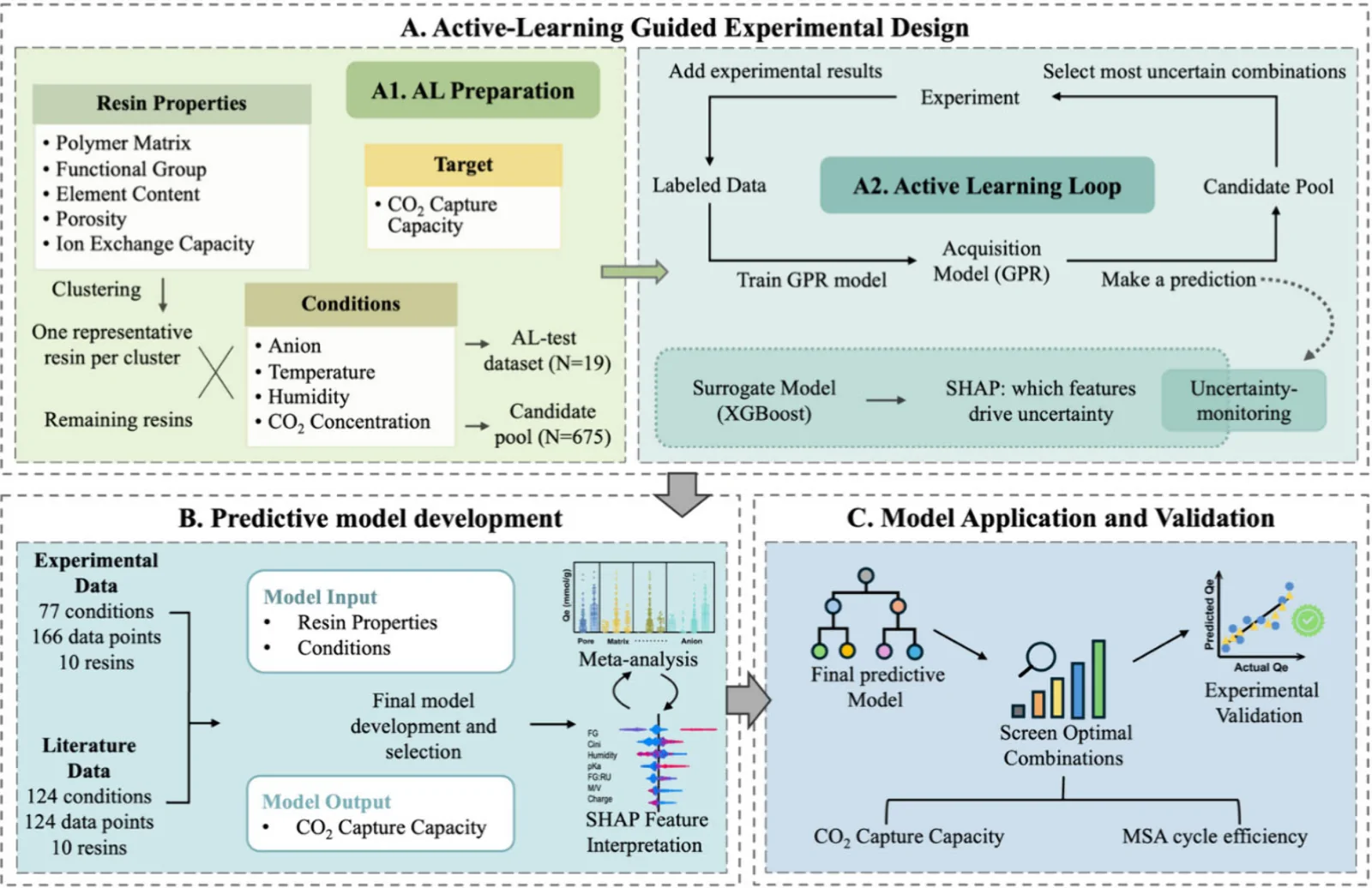
Flowchart of this research from A (A1 → A2) to B and then to C. Conditions refer to distinct experimental conditions defined by combinations of resin type, loaded anion, temperature, humidity, and CO_2_ concentration. Data points represent individual experimental measurements, including replicate measurements under the same conditions. In A2, the upper panel shows the closed AL loop used for experimental selection: a GPR model was trained on labeled data, used to predict uncertainty across the candidate pool, and the most uncertain combinations were selected for subsequent experiments and added back to the labeled dataset. The lower dashed box in A2 denotes a post uncertainty-monitoring analysis, in which the AL-derived uncertainty values were modeled with a surrogate model and interpreted using SHAP to identify features associated with high uncertainty.

## 2 Methods

### 2.1 Data Collection

We collected relevant results from two sources: internal laboratory experiments and the existing literature designated as the experimental dataset and literature dataset, respectively. This data acquisition focused specifically on MSA for CO_2_ capture using resin adsorbents (Figure S1). We first searched relevant keywords (e.g., “CO_2_ moisture swing adsorption,” “resin CO_2_ capture,”) across reputable databases, followed by secondary screening (Text S1). Literature data were manually extracted, either directly from tables or from figures using WebPlotDigitizer. A unified extraction standard was applied, replicate experimental measurements were grouped under a shared experiment identifier, and each literature condition was recorded once using its reported mean. Missing values for four features were imputed using resin-type-specific means (details in Text S2). The 15 features span resin structural properties, functional-group and counterion chemistry, and environmental conditions, together with equilibrium CO_2_ uptake as the target (Table S1). The final dataset comprises 290 data points: 166 from experiments and 124 from literature extraction. The experimental measurements correspond to 77 distinct experimental conditions (with replicates grouped by expID), while each literature entry represents one condition. In total, the dataset covers 20 resins (10 characterized in-house and 10 from literature) and 201 unique experimental conditions.

### 2.2 Materials

In this study, we systematically selected 15 commercially available AER, based on the information provided by the manufacturers, representing four major classes: macroporous strong-base, macroporous weak-base, gel-type strong-base, and gel-type weak-base resins (Table S2). To maximize diversity, the selection was based on three key parameters: resin matrix, functional group, and ion exchange capacity (IEC). We prioritized distinct matrix-functional group combinations with a wide IEC span to ensure non-redundant physicochemical properties as possible.

### 2.3 Materials Preparation and CO_2_ Adsorption Experiments

#### 2.3.1 Resin Preparation

Strong-base and weak-base resins require different pre-processing methods due to distinct initial forms (details in Text S3). To introduce target counterions (OH^–^, CO_3_^2–^, PO_4_^3–^), the resins were packed into columns and subjected to dynamic ion exchange using 0.5 M target solutions (NaOH, Na_2_CO_3_, or Na_3_PO_4_) until effluent titration confirmed complete chloride displacement. Post-exchange resins were rinsed extensively with deionized water and vacuum-dried for 12 h.

#### 2.3.2 CO_2_ Adsorption Experiments

The experimental system (Figure S2, details in Text S3) was initially purged with CO_2_ to a target concentration until the sensor readings stabilized. Then a weighed single layer of resin was placed in the reactor, N_2_-purged to remove ambient air, and connected to the system. The CO_2_ concentration curve was recorded until equilibrium (no significant change over 20 min). A parallel blank test without resin was conducted to decouple system factors (e.g., potential leaks). The equilibrium adsorption capacity was then calculated from the CO_2_ concentration difference between the sample and blank systems at equilibrium, normalized by the mass of the resin and system volume (Figure S3).

### 2.4 AL Framework

#### 2.4.1 Construction of Test Set and Candidate Pool

In the experimental design, each experiment involves one specific resin with determined experimental conditions: resin type, loaded anion (CO_3_^2–^, PO_4_^3–^, OH^–^), temperature (room temperature, 40 °C, 60 °C), humidity (dry, medium, wet), and initial CO_2_ concentration (400 ppm, 5000 ppm, 1%). Notably, weak-base AERs were limited to room temperature and 40°C due to lower thermal tolerance.

Since this study aimed to explore the reaction space rather than only identify optimal conditions, the AL-test set design was critical for uniform coverage, with each AL iteration progressively approaching the AL-test set distribution. After characterization (information in Table S3), 15 commercial resins were clustered according to their properties. After selecting one representative resin from each cluster, orthogonal design (Design-Expert software) was then applied to these resins across the experimental conditions, generating the fixed AL-test set.

The candidate pool (N = 675) was generated by pairing all remaining resins with every possible experimental condition, from which candidate experiments were selected in each AL iteration.

#### 2.4.2 Model Optimization and Uncertainty-Driven Design of Experiment (DOE)

Here, AL was used as an established adaptive sampling strategy to guide informative experiment selection under a limited experimental budget—it has been shown to reduce redundant sampling and reach a given accuracy with fewer data,^35^ and locate target candidates more efficiently in closed-loop settings.^36^ We first randomly selected 19 experimental conditions as the initial training set and built a Gaussian Process Regression (GPR) model. The model then predicted the AL-test set and simultaneously evaluated the prediction uncertainty for each candidate combination. In each iteration, the combinations with the highest uncertainty were prioritized for experiments, and the results were updated into the training set to retrain the model. This process was repeated when each iteration produced substantial gains in AL-test set R^2^ and was terminated once the marginal improvement dropped sharply, balancing model accuracy against the considerable cost of additional experiments. For practical efficiency, a batch-mode strategy was adopted, selecting multiple experiments per iteration, with the number of experiments per iteration varying based on prediction results (details in Text S4).

#### 2.4.3 Monitoring Iterative Learning Progress

Following each iteration, we monitored the model’s learning progress through two complementary approaches. First, we visualized the predictive uncertainty distribution across the candidate pool using principal component analysis (PCA)-based dimensionality reduction. The prediction uncertainty for all candidate combinations was mapped onto this space, generating a 3D uncertainty landscape that reveals how model confidence evolves as new data are incorporated (details in Text S5). Second, to attribute the source of uncertainty to features, we employed a surrogate XGBoost model trained on GPR-predicted uncertainty values for all candidate experiments (details in Text S5). These two analyses were intended as illustrative diagnostics for monitoring the source and distribution of the model’s predictive uncertainty during AL. Shapley additive explanations (SHAP) values quantified each feature’s contribution, enabling tracking of which features were associated with the model’s uncertainty across iterations.

### 2.5 Meta-Analysis

The literature and experimental datasets were merged for meta-analysis, aiming to understand the factors influencing CO_2_ capture from a broader data perspective.

#### 2.5.1 Univariate Analysis

The relationship between each feature and adsorption capacity was preliminarily examined using appropriate visualization methods. For categorical features, box plots compared capacity distributions across categories, with significance assessed by non-parametric tests (the Mann–Whitney U test for two-category comparisons and the Kruskal–Wallis test for multi-category comparisons). For continuous features, scatter plots with fitted trend lines visualized relationships, with p-values verifying significance.

#### 2.5.2 Stratified Analysis

Commercial AER data inherently exhibit non-orthogonality and sparsity, with certain features (e.g., resin matrix type and functional group type) being intrinsically confounded. To disentangle these effects, scatter plots were color-coded by confounding categorical variables, and category-specific trend lines were fitted and statistically assessed. This stratification enabled evaluation of whether observed trends persisted across subgroups or were artifacts of confounding.

A feature correlation matrix using metrics appropriate for each variable type (Text S6) was constructed to identify multicollinearity and feature interactions, informing subsequent model development.

### 2.6 Model Development and Interpretation

The dataset was split into training (80%) and testing (20%) sets. Replicate measurements under the same intended experimental conditions—resin, counter-anion, temperature, humidity, and initial CO_2_ concentration—were assigned a common group identifier (expID). In this way, replicates of the same condition were always kept within the same fold and never split between training and testing, preventing data leakage. This splitting was repeated 10 times with different random states to ensure reproducibility and robustness. Categorical features were handled via one-hot encoding. Five common ML models—random forest (RF), light gradient boosting machine (lightGBM), extreme gradient boosting (XGBoost), categorical boosting (CatBoost), and multi-layer perceptron (MLP)—were trained and compared. Random Search combined with 5-fold cross-validation was employed for hyperparameter tuning (tuned hyperparameters in Table S4). Model performance was evaluated on the testing sets using the coefficient of determination (R^2^), mean absolute error (MAE), and root mean squared error (RMSE), with separate assessments on the experimental, literature, and combined datasets to examine the influence of different data sources. Additionally, a leave-one-anion-out cross-validation was conducted to assess generalization capability across anion types (details in Text S9).

To interpret model predictions, SHAP analysis using the best-performing model was employed to quantify each feature’s contribution, with the one-hot components of each categorical feature summed into a single value per feature and three correlated functional group density descriptors combined for the interaction analysis (details in Text S10). Recognizing that analyzing individual feature contributions in isolation may overlook complex interactions, we further conducted SHAP interaction network to investigate synergistic or antagonistic effects between features, providing insights into material design-performance relationships. Reporting followed the EMBRACE checklist for environmental ML^37^ (a completed checklist in the Supporting Information).

### 2.7 Model Application and Experimental Validation

To demonstrate the best-performing model’s practical applicability, we designed two DAC application scenarios: (a) maximum adsorption capacity—for single-pass processes aiming to maximize capture efficiency, the objective was to identify conditions achieving the highest equilibrium adsorption capacity; (b) maximum moisture swing efficiency—for cyclic processes, the relevant metric is the equilibrium capacity between dry and humid conditions, which serves as an equilibrium-derived proxy for the maximum CO_2_ capacity change under a humidity swing and thus indicates the regeneration potential.

Using this model, we predicted adsorption capacity for the 15 commercial resins across a humidity range of 10–90% RH under ambient conditions (25°C, 400 ppm of CO_2_). The anion types evaluated were limited to OH^–^, CO_3_^2–^, and PO_4_^3–^. Based on these predictions, optimal resin-anion combinations were selected for each scenario. The recommended combinations were then experimentally measured after final model training, and the individual measurements were compared with predictions from the 10 random state models to prospectively validate model accuracy.

## 3 Results and Discussion

### 3.1 Collected Data Summary

After related papers were identified (details in Figure S1), the curated dataset comprises 124 distinct conditions, covering 10 AER loaded with 10 counter anions. However, the literature dataset exhibits highly skewed distribution: the data are concentrated on IRA900 resin (48 data points) and CO_3_^2–^ (74 data points), while weak-base AERs are significantly under-represented, contributing only 6 data points. To mitigate this data sparsity and broaden the coverage, new experiments were necessary to populate the under-sampled regions.

### 3.2 AL Assessment

We acquired high-value data by employing the AL strategy to optimize our experimental design. After data cleaning and the exclusion of inconsistent replicates with significant deviations, we obtained a total of 77 independent experimental conditions yielding 166 data points including replicates. Of these, 18 conditions originated from the AL-test set, while 59 were generated through five design cycles: 19 conditions for initial model training, followed by 6, 15, 7, and 12 conditions added in subsequent iterations.

#### 3.2.1 Iterations and Model Performance

The 15 commercial resins were grouped in six distinct clusters, as visualized in Figure S5 and supported by multiple cluster-validity methods (Figure S4). One representative resin from each cluster was selected, yielding a fixed AL-test set of 18 conditions (Table S5) with uniform coverage of the entire experimental design space.

In the first round, the model was trained using 19 initial conditions and exhibited suboptimal performance, with *R* ^2^ values of −0.016 for the training set and −0.211 for the AL-test set (Figure 2a). Given that the fixed AL-test set represents the design space through uniform sampling, these negative R^2^ values indicate that the initial model failed to capture meaningful patterns on the overall response surface. To guide the next iteration, the GPR model quantified the prediction uncertainty for each candidate, where high predictive uncertainty signifies a lack of model knowledge. The uncertainty surface exhibited a broad flat plateau of comparatively low uncertainty, punctuated by a singular high-uncertainty peak (Figure 2c, iteration 1). Given the extremely poor predictive performance (R^2^ = −0.211) from only 19 conditions, this widespread “low uncertainty” did not reflect true predictive confidence. Instead, the initial model suffered from insensitivity to feature variations and oversmoothing, a known phenomenon in GPR under data sparsity.^38,39^ In this regime, hyperparameter optimization tends to maximize the marginal likelihood by selecting overly smooth response functions, treating unseen variations as noise and thus obscuring the complex structures within the plateau regions.^38^ The high-uncertainty peak was the exception: it represented a region (MN100, a macroporous weak base resin) with properties far outside the training data range, correctly triggering high uncertainty.^40^

**Figure 2:**
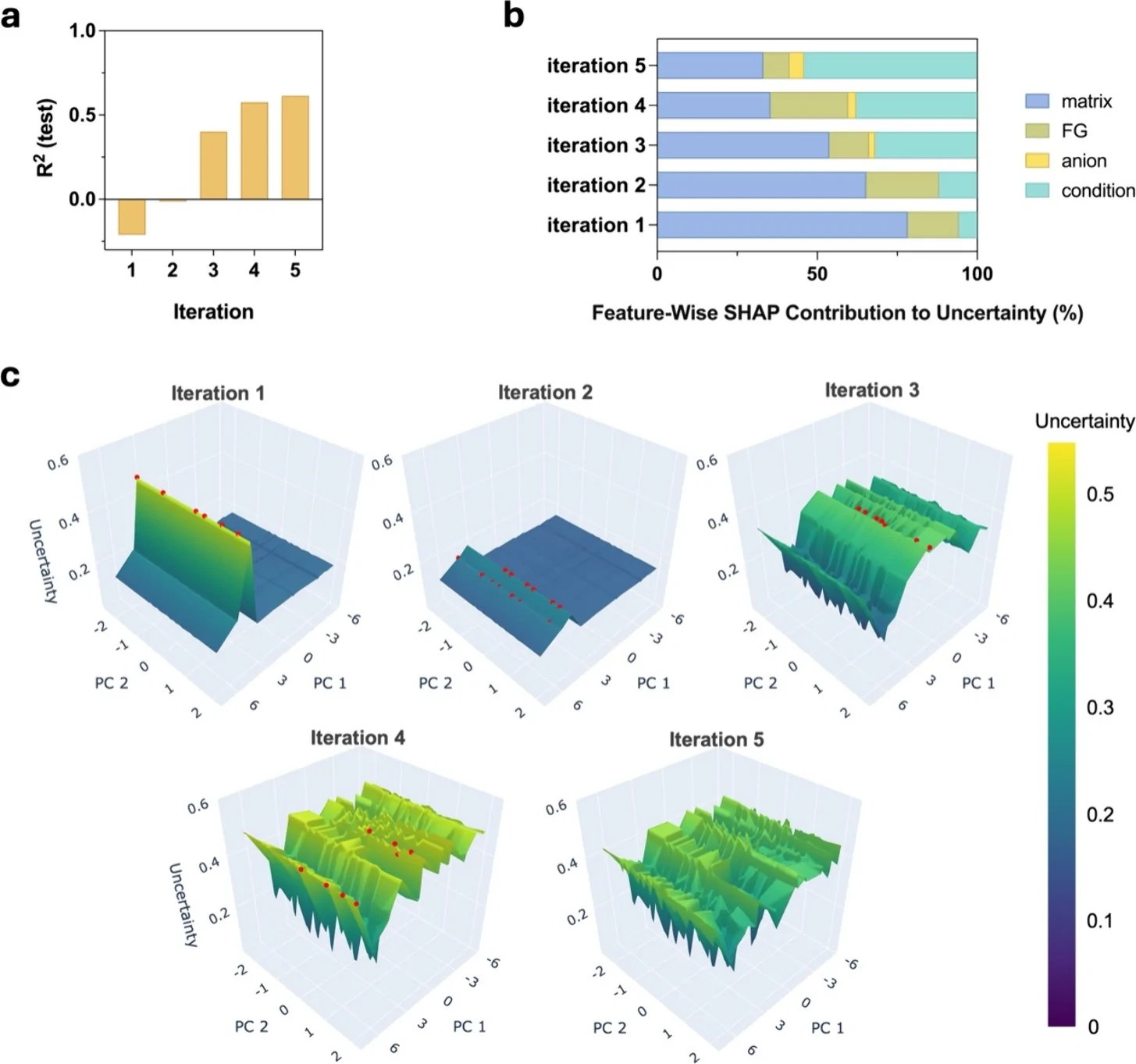
(a) Improvement in model performance on the fixed test dataset over 5 iterations. (b) Prediction uncertainty attribution to experimental features over 5 iterations, with four levels distinguished by color: matrix (dark blue), functional group (green), counter anion (yellow), and environmental conditions (light blue). (c) Visualization of prediction uncertainty response surface after each iteration throughout the AL process. The x-y panel represents the entire candidate pool (all the experiment combinations) with PCA-reduced dimensionality (PC = principal component). The surface is a 3D visualization where the z-axis and color gradient map the magnitude of predictive uncertainty. The red dots are the combinations exhibiting the highest predictive uncertainty for each iteration, which is subsequently selected for experiments and incorporated into the training set for the next iteration. This dynamic visualization tracks the specific experimental regions where model confidence increases (uncertainty decreases) as new data are integrated, enabling macroscopic monitoring of knowledge acquisition across the design space.

A surrogate XGBoost model trained on GPR-predicted uncertainty values for the entire candidate pool (N = 675, fixed throughout iterations as the completed experiments remained in the pool) attributed uncertainty to specific experimental parameters. As shown in Figure 2b, over 75% of the uncertainty in the first round was attributed to the matrix properties, with the BET surface area being predominant (Figure S6). Consequently, the next iteration targeted candidates with the highest predicted uncertainty, exclusively concentrating on MN100, which possesses a BET of 793 m^2^/g, significantly larger than the resins in the initial dataset (typically <0.2 or 20 m^2^/g). A 6-run orthogonal design was executed under varying loaded anions, temperatures, humidity levels, and initial CO_2_ concentration.

After incorporating the new data, the second-round model showed improved R^2^ values of 0.448 (training set) and −0.001 (AL-test set). Although the AL-test set R^2^ remained near zero, this improvement indicates a transition from erroneous extrapolation to capturing the global baseline, marking the onset of valid learning. The uncertainty surface of second iteration (Figure 2c, iteration 2) showed decreased uncertainty in MN100 region, although it still remained the peak of the surface. As shown in Figure S6, the dominant parameters contributing to uncertainty were BET, N%, and the polymer matrix type. For the next iteration, 6 supplementary combinations for MN100 and 9 combinations for FPA54 were selected. The inclusion of FPA54 was justified by its phenol-formaldehyde polycondensate matrix—diverging from the polystyrene or polyacrylic matrices in the existing dataset—and N% of 13%, which is different from the ∼4% prevalent in the previous resins.

In the third round, the model performance improved significantly, with *R* ^2^ values of 0.954 and 0.401 on the training and test sets, respectively. The uncertainty surface (Figure 2c, iteration 3) showed further decrease in the MN100 region while beginning to show finer structures, suggesting transition from local to global awareness of the feature space. This was corroborated by a significant increase in R^2^. Feature contributions became more balanced: matrix dominance dropped from >75% to ∼50%, while the importance of operating conditions rose, no longer overshadowed by material properties. Guided by the identified key features (BET, T, Vp, and humidity), resin FPA98 was selected for the next iteration. It has a polyacrylic matrix with a low BET (< 0.2 m^2^/g) and Vp (< 0.2 cm^3^/g), sparsely represented in the initial dataset. Seven experimental conditions for FPA98 were targeted at room temperature and dry-to -medium humidity level; these condition pairs had not been previously explored on similar resins, indicating that we are now probing unexplored intersections between specific material attributes and environmental conditions.

In the fourth round of model training, the model demonstrated a steady improvement with the AL-test set *R* ^2^ rising to 0.575 (Figure 2a). Uncertainty attribution revealed significant restructuring: the contribution of matrix-related factors dropped to 35%, while that of operating conditions rose to 37% (Figure 2b), reaching near parity, alongside increasing influence from resin functional groups and anion types. Topologically, the uncertainty surface developed more jagged features (Figure 2c, iteration 4), indicating the model could now finely distinguish between different experimental conditions, moving away from generalized predictions over broad regions. Unlike previous rounds, where high-uncertainty points concentrated within a single resin type, the candidates were dispersed across multiple resin categories (Figure S7). This dispersion suggests that the model has resolved major uncertainty gaps regarding material types and has transitioned into fine-grained exploration of the response surface. The next iteration designed 7 runs on FPA98 (targeting elevated temperatures with extreme humidity), and 5 runs on FPA51 and A510, two resins featuring a previously unexplored property combination—intermediate BET (31–35 m^2^/g) with specific ion exchange capacity (∼1.25 eq/L).

In the fifth round, the AL-test set *R* ^2^ reached 0.614. Whereas the first four iterations produced large gains in test *R* ^2^ (Δ*R* ^2^ of 0.21, 0.40, and 0.17), the fifth iteration only yielded Δ*R* ^2^ = 0.039, an abrupt drop of roughly fourfold, suggesting the model has approached a convergence plateau regarding its learning capacity within the design space. The overall uncertainty surface decrease (Figure 2c, iteration 5), indicating model convergence as it gained confidence across the entire design space. Given these reduced marginal benefits, iteration was terminated to balance accuracy and experimental cost. These results demonstrate the AL framework’s ability to efficiently target informative regions of the design space, enabling rapid model improvement with minimal experimental cost. To confirm that this improvement reflects uncertainty-driven selection rather than the addition of data alone, we compared the active-learning against random sampling from the same candidate pool in a retrospective analysis; active learning outperformed random selection at matched training sizes across the trajectory (Figure S8, Text S11).

### 3.3 Meta-Analysis

After combining literature and experimental data, we systematically divided the resin-based MSA system into four levels to analyze factors influencing performance (Figure 3a): (1) matrix structure: pore type, matrix composition, BET, Vp, and water retention. (2) Functional group properties: base type, functional group category, N%, functional group to repeat unit ratio (FG:RU), and ion exchange capacity (IEC). (3) Anion characteristics: anion species, charge number, and the pKa of the conjugate acid. (4) Environmental conditions: humidity, temperature, and CO_2_ initial concentration. The reliability of the dataset was assessed through analyses of feature distributions and significance (Figure 3), collinearity among factors (Figure S9), and the variance of replicate experimental measurements (Text S12). Due to the non-orthogonal intrinsic properties of commercial resins and literature constraints, the dataset exhibits significant feature entanglement and bias, posing substantial challenges.

**Figure 3:**
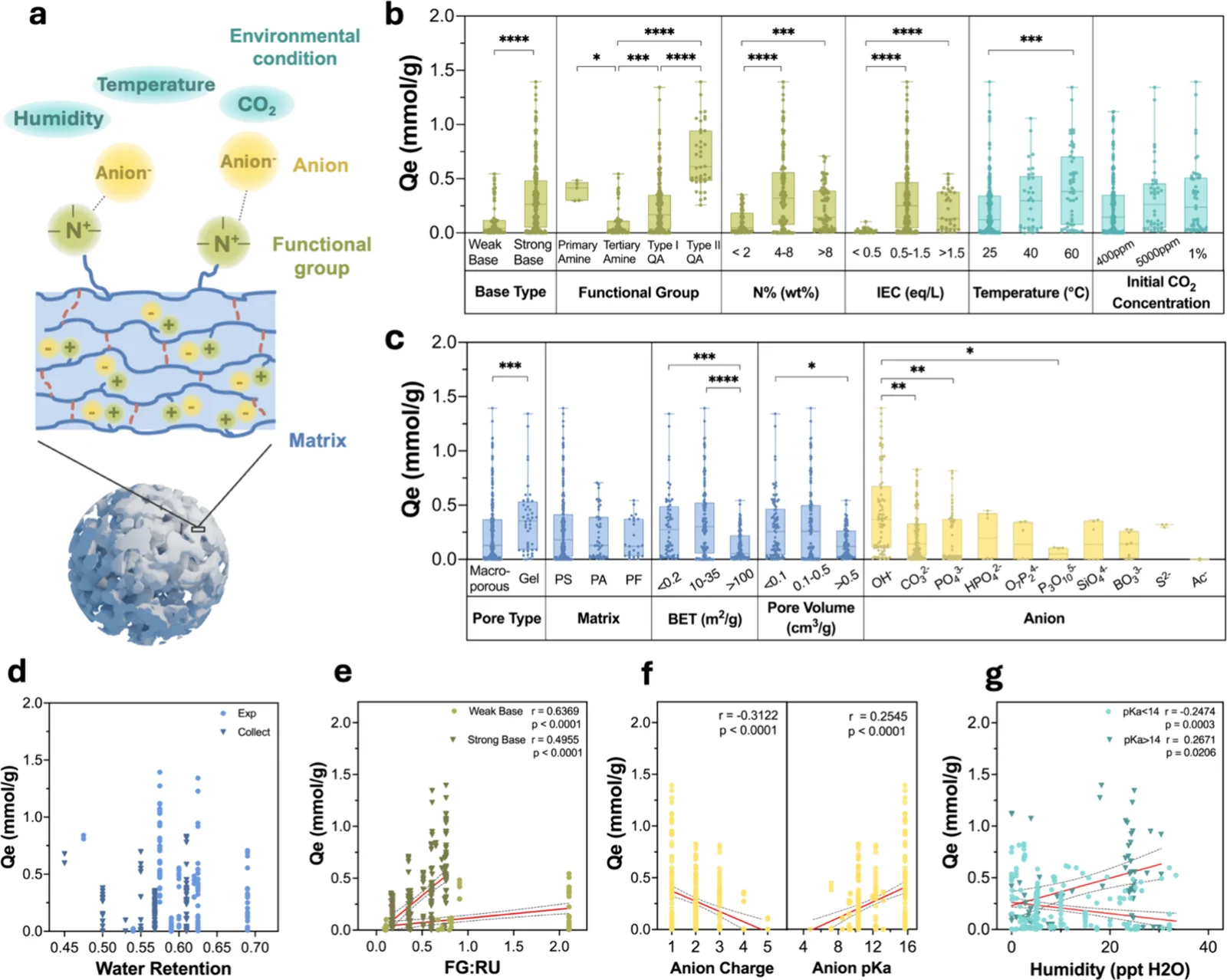
(a) Schematic of the MSA system, with four levels distinguished by color: matrix (dark blue), functional group (dark greenish yellow), counter anion (yellow), and environmental conditions (light blue). (b, c) Data distribution of various input features: base type, functional group type, N%, IEC, temperature, initial CO_2_ concentration, pore type, matrix type, BET, pore volume (Vp), and anion type. The color of each feature corresponds to its respective level’s color in Figure 3a. Asterisks (*) indicate statistical significance from nonparametric tests—Kruskal–Wallis tests for multicategory features and Mann–Whitney U tests for two-category features (****p < 0.0001; ***p < 0.001; **p < 0.01; *p < 0.05); absence of asterisks indicates no significant difference (p ≥ 0.05). (d–g) Scatter plot of water retention, FG:RU, anion charge, anion pKa and humidity. Red lines represent trend lines; gray dashed lines indicate 95% confidence intervals. Spearman’s correlation coefficients (r) and p-values are indicated.

At the matrix structure level (Figure 3c), although gel-type resins appear to outperform macroporous ones, this is likely a statistical artifact: 93% of the gel resins are in highly reactive OH^–^ form, likely masking the true influence of pore structure. No significant difference in CO_2_ adsorption capacity was observed among the three distinct polymer backbones (polystyrene PS, polyacrylic PA, and crosslinked phenol-formaldehyde (PF) polycondensate). The structural parameters exhibit nonlinear behaviors: while BET is positively correlated with CO_2_ uptake up to a threshold,^17,41^ performance diminishes at extreme values, and Vp also displays non-monotonic effects. Lastly, water retention data (Figure 3d) demonstrated consistent, trendless distributions across experimental and literature sources.

Chemical properties (functional groups and counter-anions) trends are informative yet partially obscured by data bias. In terms of base type and functional group (Figure 3b), strong-base resins—especially type II quaternary amines (QA)—generally outperform weak-base counterparts.^15^ However, the unusually high capacity for primary amines is likely a confounding artifact of predominant pairing with high-affinity anions like PO_4_^3–^ or OH^–^,^13,20,21^ rather than an intrinsic advantage of the amine groups themselves. Regarding functional group loading metrics, the ’Functional Group to Repeating Unit ratio (FG:RU)’ serves as a better predictor than N% or IEC (Figure 3b vs. e). Although N% indicates amine group quantity, it overestimates the actual functional group loading in polyacrylic resins because amide-linked groups, formed by reacting backbone ester groups with diamines,^42^ contain two nitrogen atoms per functional group. Meanwhile, manufacturer-reported IEC values are typically given as ranges, limiting accurate loading reflection. Stratifying by functional group—weak base amine vs. strong base amine—revealed a sharpened trend: FG:RU positively correlates with capacity for both, while the enhancement is more pronounced in strong-base resins (Figure 3e). This may reflect that the quaternary ammonium groups in strong base resins remain fully ionized across conditions, allowing higher functional group densities to translate more directly into enhanced adsorption capacity, whereas partial ionization in weak base resins weakens the link between functional group loading and effective adsorption sites.

On the anion front (Figure 3c), the OH^–^ ion stands out distinctively. To decipher the underlying trends, we decomposed anion identity into two critical parameters: anionic charge and the pKa of the conjugate acid (Figure 3f). First, adsorption capacity decreases with increasing anionic charge, likely driven by site-blocking: multivalent anions require multiple adjacent exchange sites for proper coordination, reducing available CO_2_ capture sites.^14,16^ Additionally, if the physical distance between functional groups is too large to be bridged by a single multivalent anion, binding becomes energetically unfavorable. This energetic barrier fosters a preference for monovalent ions.^43,44^ However, the valency influence is not straightforward. Multivalent basic ions (e.g., PO_4_^3–^) can generate protonated species (HPO_4_^2–^ and H_2_PO_4_^–^) through stepwise hydrolysis,^21^ potentially enhancing capture. In our dataset, the negative correlation suggests site-blocking dominates. Second, higher-pKa anions, as stronger conjugate bases, promote capture by undergoing greater hydrolysis in moisture. This hydrolysis releases more hydroxide ions that subsequently react with CO_2_.^22,23,25^ For OH^–^, its high affinity for CO_2_ allows direct reaction, yielding the highest adsorption performance.

Under different environmental conditions, temperature and initial CO_2_ concentration exhibited a negligible and insignificant positive correlation with capacity (Figure 2b). This primarily stems from data imbalance: for instance, at 400 ppm, the overrepresentation (84%) of inherently high-capacity strong-base resins elevates the observed mean capacity, thereby masking the true effect of initial CO_2_ concentration on adsorption performance.

Stratifying by anionic basicity helps to clarify the humidity trends (Figure 3g): weak-base anions adhere to the typical MSA mechanism (reduced capacity at high humidity), whereas strong base anions exhibit a slightly positive correlation or more complex behavior.^13,24^ Previous studies have elucidated the dry-capture/wet-release mechanisms.^13,21^ This MSA process can be generalized for a generic weak base anion A^n–^ with negative charge n, as described by eqs 1–3. In a dry environment, the anion undergoes hydrolysis (protonation), generating a hydroxide ion and free water (eq 1). This hydroxide subsequently reacts with CO_2_ to form bicarbonate (eq 2). The net CO_2_ capture process is represented by eq 3. Critically, the reaction equilibrium is governed by ionic hydration state (n and m). As stimulated in prior studies,^14,21,23,45^ CO_2_ capture is thermodynamically favorable only under dry conditions. In humid environments, water abundance alters the ionic hydration energy, shifting the equilibrium toward the reverse direction to regenerate the adsorbent and release CO_2_, which has been experimentally verified.^46,47^

$$A^{n-}\cdot nH_{2}O⇌{\mathrm{HA}}^{\left(n-1\right)-}\cdot m_{1}H_{2}O+{\mathrm{OH}}^{-}\cdot m_{2}H_{2}O+\left(n-1-m_{1}-m_{2}\right)H_{2}O$$ 1

$${\mathrm{OH}}^{-}\cdot m_{2}H_{2}O+{\mathrm{CO}}_{2}⇌{{\mathrm{HCO}}_{3}}^{-}\cdot m_{3}H_{2}O+\left(m_{2}-m_{3}\right)H_{2}O$$ 2

$$A^{n-}\cdot nH_{2}O+{\mathrm{CO}}_{2}⇌{\mathrm{HA}}^{\left(n-1\right)-}\cdot m_{1}H_{2}O+{{\mathrm{HCO}}_{3}}^{-}\cdot m_{3}H_{2}O+\left(n-1-m_{1}-m_{3}\right)H_{2}O$$ 3

In contrast, OH^–^ exhibited a positive correlation with humidity (Figure 3g). However, this trend may be influenced by data imbalance, as a few high-capacity data points at high humidity levels could disproportionately affect the fitted slope, requiring further validation.

In summary, the MSA system exhibits multicollinearity among features (Figure S8). For instance, resin matrix types are often intrinsically tied to specific functional groups, making them impossible to decouple experimentally. Similarly, counter-anion identity was redundant with conjugate-acid pKa and valence (anion charge), since an anion’s identity essentially determines these properties; we therefore represented anions by these two physical descriptors rather than categorical identity, which also allows the model to generalize to anions beyond those in the dataset. This deep entanglement undermines traditional meta-analysis reliability, as simple statistics cannot isolate individual contribution of each factor amidst complex interactions. Consequently, we employed ML models for more rigorous multidimensional analysis.

### 3.4 Model Development

#### 3.4.1 Model Performance Comparison

After feature selection (see Figure S9), 15 key features were retained for ML model development. We evaluated the 5 ML algorithms across three datasets to quantify data source quality impact (Figure 4a). Averaged across the five algorithms, models trained solely on the literature data exhibited the poorest performance and significant instability, yielding a mean test R^2^ of only 0.414 with large fluctuations. Utilizing only AL-DOE experimental data resulted in better performance, raising the mean R^2^-test of five algorithms to 0.614 while reducing volatility. The most substantial enhancement was observed when these two sources were merged into the combined dataset, where the mean prediction accuracy across all models peaked at an R^2^ of 0.656. This suggests that AL-DOE effectively filled blind spots in the parameter space, reducing model uncertainty. This is evidenced by (1) a convergence in performance across different algorithms, and (2) minimized R^2^ fluctuations across 10 random splits, indicating enhanced robustness.

**Figure 4:**
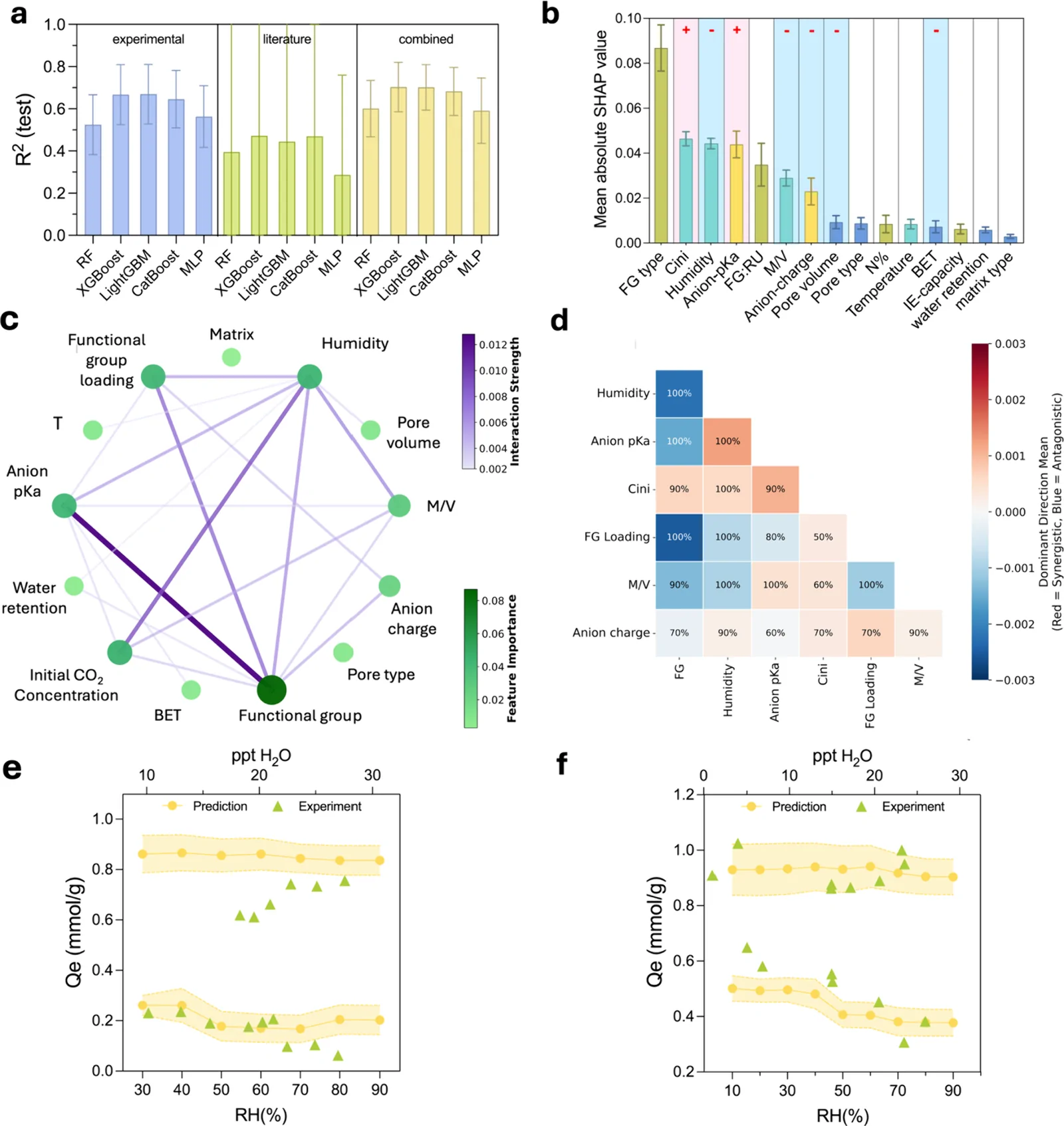
(a) Comparison of model performance across three different datasets (experimental, literature, and combined); error bars denote the standard deviation across the 10 random seeds. (b) Ranking of feature importance based on the mean absolute SHAP value. The bar colors correspond to the four feature levels defined in Figure 3a: matrix (dark blue), functional group (green), anion (yellow), and environmental conditions (light blue). A “+” sign indicates a positive contribution of the feature to the adsorption capacity, while a “–” sign indicates a negative contribution. (c) SHAP interaction network: node size/color = feature importance; edge thickness/color = interaction strength. N%, FG:RU, and IEC were grouped as “Functional Group (FG) loading” (details in Text S10). (d) Interaction directionality heatmap: color hue (red/blue) = synergistic/antagonistic effects; color intensity = magnitude; numbers = directional confidence. (e) Gel-type resins: A400 loaded with CO_3_^2–^ and HPR4100 loaded with OH^–^. (f) Macroporous resins: A510 loaded with CO_3_^2–^ and IRA910 loaded with OH^–^. In both panels, green triangles represent individual experimental measurements plotted at the actual measured humidity and Qe values. Yellow circles represent the mean model-predicted Qe values for the corresponding target humidity conditions, and the error bars indicate the standard deviation across 10 random states; the shaded band connects these standard-deviation ranges across humidity levels. Solid yellow lines are included only as visual guides connecting the predicted means.

Within the combined dataset, XGBoost and LightGBM emerged as the top performers, achieving test R^2^ values of 0.703 ± 0.117 and 0.702 ± 0.108, respectively. Although a gap exists between the training R^2^ (R^2^-train of 0.943 and 0.931, respectively) and the testing R^2^, this is typical for small datasets^48–50^ and likely reflects the limited size of the dataset, although some degree of overfitting cannot be fully excluded (Figure S10, details in Text S7). The normalized cumulative residual frequency analysis further supported the overall predictive reliability of the models (Figure S11). The model also substantially outperforms naive baselines (reference predictors that use no learned relationships) on the same splits, where the best stratified group-mean predictor – assigning each test point the mean capacity of its functional group category– reaches only R^2^ = 0.29 (Table S6). After balancing between high prediction accuracy, residual distribution and the risk of overfitting, we ultimately accepted the current model configurations and performance; LightGBM was identified as the best model and selected for the subsequent model interpretation analysis. An applicability-domain analysis was performed to further assess the model’s reliability; 97.2% of data points fell within the applicability domain (Figure S12).

#### 3.4.2 Model Interpretation

##### 3.4.2.1 Feature Importance

Due to the limited dataset size, it was crucial to validate model findings against the existing meta-analysis. SHAP analysis based on the optimal model determined feature importance and revealed whether their contributions were positive or negative.

When aggregated by 4 levels (Figure 4b, distinguished by bar color), functional group properties and ionic properties emerged as the dominant predictors, whereas resin matrix information had comparatively minor influence. Resin functional group type was identified as the most significant feature (Figure 4b). Specifically, strongly basic functional groups showed a higher capture capacity, while weakly basic groups exhibited a lower capacity. The second most important feature was the initial CO_2_ concentration (Cini), with higher concentrations leading to greater capacity. This does not conflict with the univariate analysis in meta-analysis, which looks at initial CO_2_ concentration on its own, whereas SHAP estimates its contribution once the other features are accounted for. Restricting the analysis to the DAC-relevant window (300–500 ppm) leaves the mean absolute SHAP of Cini essentially unchanged, indicating that its contribution does not arise from the high-concentration conditions (Text S8).

Following this was the humidity and ionic alkalinity; more alkaline ions yielded a higher CO_2_ capture capacity, as ions with a higher conjugate acid pKa value promote hydrolysis, facilitating OH^–^ ions generation for CO_2_ capture. To further investigate anion-specific behaviors, leave-one-anion-out cross-validation analysis revealed that OH^–^ showed relatively poor prediction accuracy compared to weak-base anions, indicating intrinsically different adsorption behavior. Unlike weak-base anions that rely on hydrolysis-mediated pathways (eqs 1–3), OH^–^ bypasses the hydrolysis step and directly captures CO_2_ (eq 4):

4 $${\mathrm{OH}}^{-}+{\mathrm{CO}}_{2}⇌{{\mathrm{HCO}}_{3}}^{-}$$

This mechanistic difference results in distinct humidity responses, confirmed by stratifying SHAP values by anion type (Figure S14). For weak-base anions (pKa < 14), SHAP values for humidity transitioned from positive to negative as humidity increased, indicating that dry conditions promote adsorption while high humidity reduces capacity—consistent with the typical MSA mechanism discussed earlier. In contrast, OH^–^ showed a weak trend, with SHAP values clustering near “0” and exhibiting small variation across humidity levels, suggesting weaker humidity sensitivity compared to weak-base anion systems within the studied experimental conditions. This differs from the apparent positive correlation observed in the meta-analysis, where high-humidity records are dominated by high-capacity OH^–^ systems and humidity is therefore conflated with anion identity when examined alone. After OH^–^ capturing CO_2_ (eq 4), the resulting monovalent bicarbonate ion theoretically lacks humidity-dependent hydrolysis free energy and thus does not undergo further hydrolysis to generate OH^–^.^20,23^ Bicarbonate and residual hydroxide ions can coexist in the dry state; bicarbonate re-enters and occupies the position of the protonated product in eq 3 (with HA^(*n*–1)–^ = HCO_3_^–^). Consequently, the system may retain some humidity sensitivity through the resulting bicarbonate-carbonate equilibrium (eq 3) upon humidification.^13,23^ Nevertheless, the overall humidity dependence of OH^–^ systems remains weaker than that of weak-base anion systems.

##### 3.4.2.2 Feature Interaction Analysis

Beyond individual feature importance, the grouped SHAP interaction analysis revealed complex nonlinear dependencies among features (Figure 4c). After grouping N%, FG:RU, and IEC as “FG loading”, the strongest interactions were dominated by functional group, anion pKa, FG loading, humidity, and initial CO_2_ concentration. Directional analysis showed three highly consistent interactions (Figure 4d). The positive and negative signs represent model-attributed non-additive effects rather than causal synergy or inhibition.

The negative anion pKa–FG interaction indicates that the effect of counter-anion basicity depends on the FG environment. In other words, a more basic anion does not provide the same additional benefit across all functional groups, because anion’s reaction and active sites jointly determine the effective CO_2_ capture environment. Similarly, the negative interactions of FG loading—with FG and with anion pKa—indicate that the effect of functional group density on capacity is not independent of the other features. Although higher loading can increase the number of exchange sites, excessively high functional group loading may induce steric hindrance or site competition, reducing overall capture efficiency. Note that the negative interaction should be interpreted as a diminishing or chemistry-dependent marginal benefit of loading, not as evidence that high loading is intrinsically unfavorable. These negative relationships indicate that practical optimization requires balancing parameters rather than pursuing extreme values of both parameters simultaneously.

The positive Cini–humidity interaction indicates that although higher humidity generally inhibit CO_2_ uptake, higher Cini may partly compensate for this decrease under humid conditions. This was also observed between pKa and humidity, indicating pKa contribution depends on humidity level. Specifically, under low humidity conditions, both high- and low-pKa anions perform relatively well. However, as humidity increases, low-pKa anions experience a more pronounced capacity decline, whereas high-pKa anions retain capacity better due to stronger hydrolysis capability. In other words, although high humidity generally reduces adsorption capacity through the MSA mechanism, high-pKa anions and CO_2_ concentration are more resistant to humidity-induced capacity loss. This finding provides practical guidance: optimal humidity ranges may vary by anion type and Cini, and matching operating conditions to specific anions can maximize capture efficiency.

Overall, the SHAP analyses convert the predictive model into practical design guidance for MSA adsorbents. FG chemistry provides the dominant control over CO_2_ uptake, while counter-anion alkalinity determines how this capacity responds to humidity. Therefore, humidity should be optimized jointly with anion identity and FG chemistry rather than treated as an independent operating variable. These results suggest that future experiments should prioritize matched combinations of resin functionality, counter-anion chemistry, and humidity conditions to identify robust high-performing MSA systems.

### 3.5 Model Application and Experimental Validation

In practical DAC applications, different process designs face distinct challenges. To demonstrate how the model provides quantitative guidance for two DAC scenarios defined in Methods, we predicted the adsorption performance of all 15 resins across 10–90% RH. Guided by the predicted adsorption profiles (Figures S15 and S16), optimal combinations were selected for experimental verification.

For gel-type resins (Figure 4e), A400 loaded with carbonate ions exhibited the highest effective CO_2_ working capacity (the predicted difference in equilibrium capacity between two different humid conditions). This is evidenced by the steep slope of the predicted capacity-RH curve, indicating large capacity differences across humidity levels, which is desirable for moisture-swing cycling. In terms of maximum equilibrium capacity, HPR4100 loaded with OH^–^ showed the highest predicted values. For macroporous resins (Figure 4f), A510 loaded with carbonate ions and IRA910 loaded with OH^–^ were identified as the corresponding top candidates. These two recommendations address different objectives: The carbonate-loaded resins are recommended for moisture-swing cycling, while the OH^–^-loaded resins define the maximum equilibrium uptake which represents a first-pass uptake ceiling– after the initial capture, humidity cycling proceeds through the carbonate–bicarbonate equilibrium rather than through OH^–^. Leverage analysis further confirms that these combinations lie within the model’s applicability domain across the studied humidity range (Figure S12). Prospective experimental validation showed that the measured capacities generally followed the predicted capacity ranges and humidity-dependent trends, and the overall predicted versus measured agreement is summarized by the parity plot (Figure S17).

Notably, gel-type resins are not recommended for dry conditions (<30% RH), where dehydration-induced shrinkage obstructs CO_2_ diffusion pathways to active sites.^51^ Conversely, macroporous resins, characterized by higher cross-linking density and structural rigidity,^52^ maintain permanent porosity regardless of humidity.^53^ This distinction is corroborated by our meta-analysis (Figure S18), revealing a significant humidity dependency for gel-type resins, where capacity drops significantly in dry environments, in contrast to the stable macroporous performance.

### 3.6 Limitations and Outlook

The predictive performance is currently constrained by the limited size and coverage of the dataset rather than by model capacity and is expected to improve as more data become available. As the dataset grows, more expressive modeling strategies, including hybrid or ensemble architectures, could be explored to further improve predictive accuracy.

Counter-anions are represented by two physical descriptors, conjugate-acid pKa and valence, allowing the model to generalize beyond specific anion labels. However, this set captures only acid–base and electrostatic characteristics; extending it with properties such as hydration energy, ionic radius, and proton affinity, and broadening the range of anion chemistries in the training data, would improve transferability to anions that differ substantially from those studied here.

Our framework predicts the equilibrium CO_2_ uptake, measured here in a closed-chamber batch system in which the gas-phase CO_2_ concentration decreases during adsorption until equilibrium is reached. The reported values thus represent batch-equilibrium capacities from the stated initial concentration. More broadly, while equilibrium capacity provides a useful, thermodynamically grounded indicator of the achievable moisture swing, it does not by itself determine practical MSA performance, which additionally depends on adsorption/desorption kinetics, cyclic stability, regeneration efficiency, water management, mechanical durability, and process-level energy consumption. The present study therefore characterizes the equilibrium capacity of the system rather than its full operational performance. Future work could extend the framework to these process-level metrics, for example, by incorporating kinetic and cyclic-stability measurements as additional target variables, and by evaluating performance in flow-through experiments that better represent practical DAC operation. Such extension would bring the approach closer to guiding practical DAC material and process design.

## 4 Environmental Significance

Accurate predictive models are essential for optimizing adsorbent materials and process systems while elucidating structure-property relationships governing adsorption performance. Addressing the challenge of limited and imbalanced datasets in practical scenarios, this study established a data-driven AL framework that improves knowledge acquisition efficiency from limited experimental iterations. This approach enabled the development of a predictive model for AER-MSA systems spanning 20 commercial resins, achieving reasonable predictive performance despite the limited and heterogeneous dataset. Importantly, the framework provides an experimental design paradigm that strategically supplement fragmented literature datasets with targeted experiments, providing a methodological reference for similar data-scarce experimental studies.

We also integrated meta-analysis with SHAP interpretation to disentangle complex multifactorial interactions. This dual-analytical approach successfully extracted feature trends from datasets plagued by imbalances and inherent material property coupling—challenges that confound traditional univariate analysis.

Our analysis identified quaternary ammonium groups, CO_2_ concentration, humidity and anion basicity as primary determinants of capture capacity, with three feature pairs (anion pKa-functional group, CO_2_ concentration–humidity, and functional group–loading) exhibited pronounced net interaction effects. These insights provide actionable guidance for both accelerating material discovery and optimizing process operations: future optimization efforts can prioritize these key features and systematically tune the levels of relevant parameters to identify the optimal combination for enhanced performance. Additionally, the predictive model enables capacity forecasting across diverse operating conditions, supporting optimal design of DAC processes. While this work focuses on equilibrium capacity prediction, it establishes a foundation for materials screening and provides mechanistic understanding that may inform future process optimization efforts toward energy-efficient DAC technologies.

## Supplementary Material



## Data Availability

The data and the code within the present study are available within the GitHub depository at https://github.com/PYU-pub/MSA-ML.

## Notes

More details about data collection, materials, experiment setup, AL process, data mining, additional results and discussion (PDF)
