# Supplementary material for: Active Learning-Guided Optimization of Moisture Swing Adsorption for Direct Air Capture
Source: Environ Sci Technol. 2026 Sep 26;60(40):28845–57. doi: 10.1021/acs.est.6c07244 (PMC13674078; doi:10.1021/acs.est.6c07244)
Supplement: Supplementary file 1 [file es6c07244_si_001.pdf]

Supporting Information for

Active Learning-Guided Optimization of Moisture Swing Adsorption for Direct Air Capture

Peiling Yu†, Kai Zhang†, Xiaoyang Shi, Zihan Zheng†, Huichun Zhang†*

†Department of Civil and Environmental Engineering, Case Western Reserve University, Cleveland, OH 44106, United States

‡Department of Earth and Environmental Engineering, Columbia University, New York, NY 10027, United States

*Corresponding Author: Phone: (216) 368-0689; E-mail: hjz13@case.edu

The supporting information (a total of 35 pages including the cover sheet) contains: Summary: 35 pages, 12 texts, 6 tables, 18 figures.

Text S1. Article Screening

To define the scope of relevant studies, we employed the PICO framework (Fig. S1a). This framework guided our literature screening by specifying the target adsorption systems (Population), the key variables influencing adsorption performance (Intervention/Exposure), the basis for comparing results across different studies and conditions (Comparison), and the primary outcome measure to be extracted (Outcome).

Literature searches were conducted in Web of Science and Google Scholar using keywords such as "CO_2 moisture swing adsorption," "resin CO_2 capture," and "MSA performance." The screening process followed PRISMA guidelines, with detailed screening stages and the number of studies at each stage (Fig. S1b). Studies were included if they reported experimental batch adsorption data for commercial anion exchange resins with complete system parameters. Exclusion criteria included non-AER systems, DFT theoretical work lacking empirical data, reports with incomplete experimental parameters, and non-batch adsorption setups.

Text S2. Data Extraction

CO_2 adsorption capacity data were extracted from figures in the included studies using WebPlotDigitizer. All extracted data were standardized to consistent units: temperature in °C, humidity in ppt water, and adsorption capacity in mmol/g.

Resin characteristic data were obtained from multiple sources. Basic resin properties—including base strength (strong/weak), matrix porosity (macroporous/gel), matrix type, water retention, and ion exchange capacity—were obtained from manufacturer specifications. For water retention, the midpoint of manufacturer-reported ranges was used. Additional properties including BET surface area, pore volume, and nitrogen content (N%) were collected from the literature.[1-6] BET surface area and pore volume were missing for 5 of 20 resin types (21 of 290 data points, ≈7.2%); nitrogen content (N%) was missing for 6 of 20 resin types (28 data points, ≈9.7%). Missing values were confined to these four features and imputed using the mean of available data for the corresponding resin type.

The mass-to-volume ratio (M/V) was calculated from the adsorption system volume and resin mass reported in each study. When this information was unavailable, M/V was estimated

based on the experimental scale: studies conducted at the 400 ppm CO_2 level were assigned M/V = 0.05 g/L, while studies using large system volumes (e.g., ~4.5 L) were assigned M/V = 0.01 g/L. M/V was estimated in this way for 82 of 290 data points (≈28%).

The functional group to repeat unit ratio (FG:RU) was calculated to quantify functional group loading on the resin matrix. This metric considers only the repeat unit (RU) and functional group (FG) components of the resin structure. The molecular identity and weight of each component were determined from manufacturer specifications and characterization data. The molar amount of functional groups per gram of resin (n_{FG}) was calculated from the nitrogen content:

$$n_{FG} = \frac{N\%/100}{M_N}$$

where M_{N} is the atomic mass of nitrogen (14 g/mol). The molar amount of repeat units per gram of resin (n_{RU}) was then calculated as:

$$n_{RU} = \frac{1 - n_{FG} \cdot M_{FG}}{M_{RU}}$$

where M_{FG} and M_{RU} are the molecular weights of the functional group and repeat unit, respectively. The ratio FG :RU = $n_{\mathrm{FG}}/n_{\mathrm{RU}}$ represents the average number of functional groups per repeat unit.

For experiments, replicate measurements under identical conditions were all retained but assigned a shared experiment identifier (expID) to prevent replicates from being split across training and test sets. For literature data, each distinct experimental condition was recorded once using the mean value reported in the source. Extracted values were checked against physically plausible ranges; no further statistical outlier removal was applied to preserve the full range of observed behavior.

To assess inter-laboratory bias, the CO_2 uptake distributions of the in-house and literature datasets were compared within a comparable-condition subset (~400 ppm CO_2). The two sources showed no statistically significant difference (Mann-Whitney t test, $p = 0.065 > 0.05$), indicating that systematic inter-study bias is limited under comparable conditions. Combined with the inclusion of measurement conditions as explicit model features and the tracking of data source for every record, this supports the pooling of in-house and literature data for modeling.

To confirm that model performance does not rely on features containing imputed values, and that such features do not act as implicit source indicators, we performed a feature-ablation

study. Each ablation variant removed one or more imputed features (M/V, BET, Vp, N%) and we retrained the model under the identical 10-seed grouped cross-validation protocol with per-seed hyperparameter re-tuning, so that each variant is directly comparable to the full model. Removing M/V, the most heavily imputed feature, changed the test R^2 from 0.7020.108 to 0.6960.114; and removing all imputed features simultaneously (M/V, BET, Vp, N%), from 00.7020.108 to 0.6980.093. The change is within the run-to-run standard deviation (≈0.09–0.12), indicating that the model's predictive performance is not driven by imputed values or any implicit source information they might encode.

Text S3. Resin Characterization, Pre-treatment, and Experimental System Setup

Resin Characterization. Prior to analysis, all samples were washed with ethanol to remove impurities and dried at room temperature for 12 h. For elemental analysis, carbon, hydrogen, and nitrogen contents were determined using an elemental analyzer (Elementar Unicube, German). For BET and porosity analysis, specific surface area and pore volume were measured by N_2 adsorption-desorption at 77 K (Micromeritics ASAP 2460, USA). Samples were degassed at 50°C for 8 h prior to measurement. A relatively low degassing temperature was used to avoid thermal decomposition of amine functional groups, particularly the weak-base amine groups. BET surface area was calculated from the adsorption isotherm, and pore volume was determined using the BJH method. Characterization results are summarized in Table S2.

Resin pre-treatment. Strong-base resins inherently contain exchangeable chloride ions as counterions, whereas weak-base resins initially exist in free amine forms. To activate weak-base resins, all samples underwent continuous washing with 5% HCl solution under stirring for 8 h, followed by thorough rinsing with deionized water, converting free amine groups into chloride-bound species.

Experimental system. The CO_2 adsorption experiment system (Figure S2, total volume ≈ 2.6 L) comprised a hermetically sealed, climate-controlled chamber integrated with humidity and temperature regulation subsystems. System integrity was validated through leak testing under a high CO_2 concentration and a large internal–external gradient: over 11 hours, the internal CO_2 concentration decreased from 10200 to 9700 ppm (a 500 ppm drop) in the 2.59 L system,

corresponding to a leak rate of ~4.8 mol·h^{-1}. Because the leak rate is proportional to the internal–external concentration difference, leakage during the low-concentration experiments is substantially smaller. In all cases, any residual leakage and sensor drift are corrected by the parallel blank measurement. Humidity was maintained by a humidity control unit that used saturated salt solutions, water reservoirs, and silica gel desiccants, while reactor temperature was stabilized using a recirculating water bath. Gas circulation at 12 L·min^{-1} was driven by a diaphragm pump, with real-time CO_2 monitoring achieved via dual-range NDIR sensors (CM-0026 for low concentrations; CM-40831 for high concentrations; CO_2 Meter). The resin mass was adjusted according to the expected uptake (0.02–0.5 g at 400 ppm) to keep the CO_2 concentration depletion within a reasonable range while maintaining a measurable signal. Reported capacities are batch-equilibrium values measured from the stated initial CO_2 concentration, following established closed-chamber methods for moisture-swing sorbents.[1, 6]

Text S4. Batch-Mode Active Learning Strategy for Experimental Design

The initial training set of 19 experimental conditions corresponded to our preliminary experiments and provided a minimal but sufficient basis to train an initial model and initiate the active-learning loop. We used these data as the seed set because they covered the main experimental variables needed to initialize the GPR model, including different resin/anion combinations and a range of humidity and CO_2 concentration conditions. Gaussian Process Regression (GPR) was chosen as the acquisition model because it is a well-established choice for uncertainty-driven active learning[7, 8]: it provides principled, calibrated predictive uncertainty (posterior variance) directly, which is required for uncertainty-driven sampling, and is well suited to small datasets.

A batch-mode active learning strategy was adopted for practical efficiency. At each iteration, experiments were selected based on uncertainty estimates from the GPR model. This uncertainty was determined by the posterior variance output of GPR, reflecting the level of confidence the model had under the current data support: higher uncertainty indicated lower confidence in accurate prediction, whereas lower uncertainty indicated greater confidence. When high uncertainty was concentrated in a specific resin (i.e., uncertainty values across conditions

for that resin were similarly elevated), orthogonal experimental design was applied to efficiently cover the condition space for that resin within a single iteration. When high uncertainty was distributed across multiple resins rather than concentrated in one, experimental combinations were selected in descending order of predicted uncertainty. The number of experiments per iteration was not fixed and was determined by the structure of the uncertainty distribution in that iteration, rather than by a single preset value: iterations with uncertainty concentrated in two resins required more experiments than those involving a single resin to adequately characterize those regions. If a resin selected in a previous iteration still exhibited high uncertainty for unexplored conditions, supplementary experiments were conducted for that resin alongside new experiments for other resin's high-uncertainty combinations. Thus, while the identification of high-uncertainty resins and regions was driven quantitatively by the GPR uncertainty, the construction of each iteration's orthogonal design involved expert judgment in selecting the specific conditions to probe; the per-iteration counts were not set by a fixed automated threshold.

The specific resin selections and rationale for each iteration are detailed in Section 3.2.1.

Text S5. AL Process Monitoring

PCA Visualization of Uncertainty Distribution. We utilized Principal Component Analysis (PCA) to reduce the high dimensionality of the experimental feature space—including resin type, exchange ion, and experimental conditions (T, moisture, and initial CO_2 concentration)—into two principal components. The prediction uncertainty for all candidate experimental combinations was mapped onto this reduced dimensional space, providing a 3D visualization of the uncertainty distribution. This dynamic, visualized tracking demonstrates the specific experimental regions where the model's predictions become more confident (i.e., uncertainty decreases) as new data are added, thereby allowing us to monitor the model's knowledge acquisition and the evolution of its perception across the design space in a big picture.

Surrogate Model for Uncertainty Attribution. At each AL iteration, the GPR model output prediction uncertainty for all remaining experimental conditions in the candidate pool. We then constructed an interpretable XGBoost regression model (retrained at each iteration) with all candidate experiments' uncertainty values as the target variable (Y) and the corresponding

experimental features as input variables (X):

The surrogate XGBoost model was trained to capture the relationship between experimental features and prediction uncertainty. Shapley additive explanations (SHAP) values were then calculated for each feature, quantifying its contribution to the predicted uncertainty. Features with high SHAP values indicate conditions where the GPR model remains uncertain—representing knowledge gaps that the model has "yet to learn". By tracking the evolution of SHAP-based feature attributions across iterations, we linked uncertainty reduction to specific experimental features and tracked how the model's predictive uncertainty was distributed across the experimental space during AL. We emphasize that this analysis is an illustrative diagnostic for interpreting the source of the GPR's uncertainty.

1. *Rationale.* We used a tree-based surrogate rather than applying model-agnostic SHAP directly to the GPR because the GPR posterior uncertainty does not admit an efficient exact-SHAP formulation; kernel-based SHAP on the GPR across the full candidate pool (N = 675) at every iteration would be computationally expensive and subject to sampling variance. Training an XGBoost surrogate on the GPR-predicted uncertainty, using the same input features as the GPR, allows fast, stable, and exact TreeSHAP attribution.
2. *Surrogate fidelity.* The surrogate reproduced the GPR-predicted uncertainty with high fidelity across all iterations: the mean cross-validated R^2 was near-perfect in the early iterations (0.99999 and 0.9999 for iterations 1 and 2) and remained high thereafter (0.976, 0.952, and 0.981 for iterations 3, 4, and 5). This high fidelity is expected, as the GPR posterior uncertainty is a smooth, deterministic function of the input features—depending on the position of each feature combination relative to the training data rather than on noisy measured labels—and is therefore well reproduced by a flexible surrogate. The modest decrease in later iterations is consistent with the increasingly complex uncertainty landscape as more data are incorporated. These results confirm that the surrogate faithfully captures the GPR uncertainty landscape, so the SHAP attributions reliably reflect the sources of GPR uncertainty. These results indicate that the surrogate closely reproduces the GPR uncertainty estimates, supporting the use of surrogate-based SHAP analysis to interpret the major feature contributions to GPR uncertainty.

Shapley additive explanations (SHAP) values were then calculated for each feature, quantifying its contribution to the predicted uncertainty. Features with high SHAP values indicate conditions where the GPR model remains uncertain, representing knowledge gaps that the model has yet to learn.

Text S6. Correlation Analysis for Mixed-Type Variables

We employed Spearman rank correlation for continuous-continuous pairs (capturing monotonic relationships without assuming linearity), Cramér's V for categorical-categorical pairs (measuring association strength between nominal variables), and Correlation Ratio (η) for categorical-continuous pairs (quantifying the proportion of variance in the continuous variable explained by categorical grouping). These metrics were integrated into a unified correlation heatmap to provide a holistic view of feature interdependencies. Notably, Spearman coefficients range from -1 to 1 (indicating direction and strength), whereas Cramér's V and Correlation Ratio range from 0 to 1 (indicating strength only). Result was shown in Figure S8.

Text S7. Model Evaluation Protocol, Assessment of Overfitting and Resin-level Generalization

Evaluation protocol. Five-fold cross-validation was chosen as a bias–variance compromise for a dataset of this size: fewer folds shrink the training set and increase bias, whereas more folds (or leave-one-out) reduce each validation fold further and increase variance. Five folds also leave enough independent groups per fold under grouped partitioning (GroupKFold by expID). No single partition was relied upon: the procedure was repeated across 10 random seeds with results reported as mean ± standard deviation, so averaging reduces the variance of the estimate while the reported standard deviation quantifies the remaining uncertainty. Final performance was assessed on an independent held-out test set, likewise averaged over the 10 seeds.

Assessment of Overfitting. Overfitting was controlled during model development through

regularization and complexity-limiting hyperparameters (L1/L2 regularization, maximum tree depth, minimum child samples, and subsampling), grouped cross-validation (GroupKFold by expID), and evaluation on an independent test set averaged over 10 random seeds. The learning-curve analysis (Figure S10) shows that cross-validated performance continues to improve with training-set size, indicating that the train-test gap is driven substantially by the limited dataset size; some degree of overfitting nonetheless cannot be fully excluded.

Text S8. Feature importance within the DAC-relevant concentration subset

To test whether the high SHAP ranking of the initial CO_2 concentration (Cini) is driven by the 5000 ppm and 1% conditions rather than by behavior near the DAC range, we recomputed mean absolute SHAP within the subset of samples with Cini between 300 and 600 ppm (99 datapoints per seed, 34.1% of all datapoints). Importances were averaged within each seed and then across the 10 seeds, so that each seed contributes equally.

Cini within this subset was similar to its full-dataset contribution (mean absolute SHAP 0.0532 versus 0.046), remaining the same order in rank. In the DAC range (300-500 ppm), the top 5 important features are as follow: FG, Cini, humidity, anion pKa. M/V. This ranking is the same as the fullest importance ranking except M/V. The larger contribution of M/V in this regime is consistent with the smaller absolute amount of gas-phase CO_2 available at low concentration, which makes the capacity more sensitive to the solid-to-gas ratio.

The overall ordering is otherwise stable between the two analyses: no feature moves by more than two rank positions, and the four least important features (BET, IE-capacity, water retention, matrix) remain almost unchanged at the bottom. Within the DAC-relevant subset, functional group is by a clear margin the dominant contributor (mean absolute SHAP 0.0991, roughly 2 times that of Cini), indicating that under DAC-relevant conditions the capacity is governed primarily by the resin chemistry rather than by the operating concentration.

Notably, these analyses use SHAP values from the final model applied to the subset samples; the model itself was not retrained on the subset, since the aim is to characterize the contribution of each feature within the deployed model rather than to fit a separate low-concentration model.

Text S9. Leave-One-Anion-Out Cross-Validation: Methods and Results

To rigorously assess the model's generalization capability on entirely new, unseen data, we adopted a unique cross-validation strategy: leave-one-anion-out. Anion type was selected for this validation because it is a key categorical feature governing adsorption mechanisms (e.g., OH^- versus weak-base anions exhibit distinct capture pathways), and predicting performance for new anion types represents a practically relevant generalization scenario. The seven anion types in this study also provided a suitable number for leave-one-out validation. In this approach, we used data corresponding to one anion type as a complete test set, while the remaining anion data served as the training set. This method allowed us to more strictly evaluate the model's accuracy in predicting unseen datasets and, specifically, to assess its ability to generalize to new anion information.

The result (Fig. S7) showed that the model achieved satisfactory predictive performance for most anions, with SiO_4^{2-} and $P_2O_7^-$ showing the best prediction accuracy. The CO_3^{2-} ion exhibited the poorest predictive performance. This is primarily attributed to a significant loss of critical information in the training set when CO_3^{2-} was removed, as its data entries accounted for 43% of the entire dataset, severely impairing the model's ability to learn universal patterns. The OH^- ion, being a strong-base anion, also showed poor prediction accuracy. This finding indicates that the adsorption behavior of the OH^- ion is intrinsically different from that of the weak-base anions.

Text S10. Grouped SHAP analysis

To maintain a consistent interpretation level, SHAP values for one-hot encoded categorical descriptors were aggregated back to their original features by summing the signed SHAP values of the corresponding dummy variables. For the interaction analysis, N%, FG:RU, and IEC were additionally grouped as “FG loading” because they describe related aspects of functional group density and are mutually correlated. Grouping was performed only at the interpretation stage using SHAP values from the same final LightGBM model; the model was not retrained using the merged descriptors. Cini and M/V were retained as separate descriptors because they represent

physically distinct quantities, namely gas-phase CO_2 concentration and solid-to-gas ratio. All grouped SHAP results were therefore interpreted as model-conditional feature attributions, not causal effects.

Text S11. Retrospective comparison of active learning against random selection

To assess whether the improvement in test-R^2 reflects uncertainty-driven selection rather than the addition of data alone, we compared the actual active-learning trajectory against random selection from the full candidate pool in a retrospective in-silico analysis. Random selection used the same batch sizes, the same GPR model-selection procedure, and the same independent AL-test set as the active-learning run, and was repeated over 20 random draws. Because the randomly selected candidates were not experimentally measured, their Qe values were assigned using predictions from the final LightGBM model; we therefore describe this as an in-silico reference rather than an independent experimental validation, so the comparison should be read as indicative rather than as an unbiased benchmark.

The actual active-learning trajectory improved test-R^2 more rapidly than the mean random trajectory, most clearly at intermediate training sizes (Figure S8).

Text S12. Replicate measurement variability

Repeated in-house measurements under each condition (expID) were used to quantify label noise. Of the 77 distinct experimental conditions, 67 were measured in replicate (156 measurements in total), the within-condition coefficient of variation of Qe had a median of 7.7% and a mean of 0.024 mmol/g in absolute standard deviation. A small number of conditions with low absolute Qe show larger CV values due to the small denominator, but the low absolute standard deviation indicates that replicate measurements are consistent. Literature entries are single-point values and were not included in the above calculation.

Table S1. Summary of the input features and prediction target used in the machine learning model. For each feature, the physical meaning, unit, and variable type (continuous or categorical) are listed. The target variable is the CO_2 uptake (Qe). Counter-anions are represented by physical descriptors (conjugate-acid pKa and valence) rather than by categorical identity.

**Feature**	**Physical meaning**	**Unit**	**Type**
pore	Matrix pore architecture (macroporous/gel)	—	Categorical
matrix	Polymer matrix type	—	Categorical
FG	Functional group type (primary/tertiary amine, type I/II quaternary amine)	—	Categorical
water_retention	Water retention of resin	%	Continuous
BET	BET surface area	m^2/g	Continuous
Vp	Pore volume	cm^3/g	Continuous
N%	Nitrogen content	wt%	Continuous
FG:RU	Functional group to repeat unit ratio	—	Continuous
IE-capacity	Ion exchange capacity	mmol/g	Continuous
anion_charge[a]	Counter-anion valence	—	Continuous
anion_pKa[a]	the pKa of the conjugate acid of the counter-anion	—	Continuous
T	Temperature		Continuous
humidity	humidity	ppt water	Continuous
Cini	Initial CO_2 concentration	%	Continuous
M/V	Mass to volume ratio	g/L	Continuous
Qe (output)	CO_2 uptake	mmol/g	Continuous (target)

[a] Counter-anion identity ($OH^-/CO_3^{2-}/PO_4^{3-}$, categorical), used during the exploratory active-learning stage, was found to be collinear with these two descriptors in the meta-analysis and thus was replaced by conjugate-acid pKa and valence (anion charge) in the final model to reduce redundancy and enable generalization to unseen anions.

Table S2. 15 commercial resins: properties and manufacture information summary.

No.	Name	Base	Structure	Functional Group	Manufacturer
1	A420S	Strong Base	Gel Polystyrene-DVB	Type I QA	Purolite
2	A400	Strong Base	Gel Polystyrene-DVB	Type I QA	Purolite
3	HPR4100	Strong Base	Gel Polystyrene-DVB	Type II QA	DuPont
4	MN100	Weak Base	Macro Polystyrene-DVB	Tertiary Amine	Purolite
5	MN150	Weak Base	Macro Polystyrene-DVB	Tertiary Amine	Purolite
6	FPA51	Weak Base	Macro Polystyrene-DVB	Tertiary Amine	DuPont
7	A110	Weak Base	Macro Polystyrene-DVB	Primary Amine	Purolite
8	A100	Weak Base	Macro Polystyrene-DVB	Tertiary Amine	Purolite
9	IRA96	Weak Base	Macro Polystyrene-DVB	Tertiary Amine	DuPont
10	A500PS	Strong Base	Macro Polystyrene-DVB	Type I QA	Purolite
11	FPA54	Weak Base	Macro- Crosslinked phenol-formaldehyde polycondensate	Tertiary Amine	DuPont
12	IRA910	Strong Base	Macro Polystyrene-DVB	Type II QA	DuPont
13	A510	Strong Base	Macro Polystyrene-DVB	Type II QA	Purolite
14	IRA458	Strong Base	Gel-Polyacrylic	Type I QA	DuPont
15	FPA98	Strong Base	Macro-Polyacrylic	Type I QA	DuPont

Table S3. Physicochemical properties of the selected 15 commercial AERs characterized and used in the active-learning process.

No.	Resin	Pore	Polymer matrix	Functional group	BET(m^2/g)	Pore volume (cm^3/g)	N%	Ion exchange capacity (eq/L)	Cluster
1	A420S	gel	PS-DVB	Type I QA	0.1638	0.052×10^{-3}	4.526	0.8	1
2	A400	gel	PS-DVB	Type I QA	0.2305	0.046×10^{-3}	4.0915	1.3	1
3	HPR4100	gel	PS-DVB	Type II QA	0.1508	0.024×10^{-3}	4.511	1.2	1
4	MN100	macro	PS-DVB	Tertiary Amine	793.468	0.272	1.099	0.2	2
5	MN150	macro	PS-DVB	Tertiary Amine	914.132	0.631	0.959	0.2	2
6	FPA51	macro	PS-DVB	Tertiary Amine	35.904	0.204	6.863	1.3	3
7	A110	macro	PS-DVB	Primary Amine	30.155	0.282	8.748	2	3
8	A100	macro	PS-DVB	Tertiary Amine	32.326	0.205	5.332	1.3	3
9	IRA96	macro	PS-DVB	Tertiary Amine	32.857	0.153	6.384	1.3	3
10	A500PS	macro	PS-DVB	Type I QA	13.967	0.047	4.740	1.1	3
11	FPA54	macro	Crosslinked PF	Tertiary Amine	101.857	0.833	13.063	1.8	4
12	IRA910	macro	PS-DVB	Type II QA	20.155	0.274	4.719	1	5
13	A510	macro	PS-DVB	Type II QA	30.665	0.314	4.294	1.2	5
14	IRA458	gel	PA	Type I QA	0.161	0.102×10^{-3}	10.271	1.25	6
15	FPA98	macro	PA	Type I QA	0.122	0.098×10^{-3}	10.157	0.8	6

Table S4. Hyperparameters of the five ML models, tuned by randomized search with grouped inner cross-validation (GroupKFold by expID). Values shown are the most frequent configurations selected across the 10 random seeds. Hyperparameter tuning used only the training data via cross-validation; the independent held-out test set was not used for tuning and served only for final evaluation.

Cluster	Key hyperparameters (most frequent across 10 seeds)
Random Forest	n_estimators=200, min_samples_split=4, min_samples_leaf=3, max_features=0.7, max_depth=6
XGBoost	Subsample=0.8, reg_lambda=1, reg_alpha=0.5, n_estimators=270, min_child_weight=7, max_depth=6, learning_rate=0.06, colsample_bytree=0.8
LightGBM	Subsample=0.8, reg_lambda=3, reg_alpha=0.5, n_estimators=150, min_child_samples=6, max_depth=6, learning_rate=0.07, colsample_bytree=0.8
CatBoost	Rsm=0.5, random_strength=9, learning_rate=0.06, l2_leaf_reg=10, iterations=270, depth=5, bagging_temperature=7
MLP	learning_rate_init=0.01, hidden_layer_sizes= (128, 64), alpha=0.01, activation= “relu”

Table S5. Experimental conditions comprising the fixed AL-test set (18 conditions across 6 representative resins).

Cluster	Resin	Anion	Temperature	Humidity Level	Initial CO_2 concentration
1	A420S	OH^-	40	wet	400ppm
1	A420S	CO_3^{2-}	60	dry	5000ppm
1	A420S	PO_4^{3-}	25	medium	1%
2	MN150	OH^-	30	wet	1%
2	MN150	CO_3^{2-}	25	dry	400ppm
2	MN150	PO_4^{3-}	40	medium	5000ppm
3	A110	PO_4^{3-}	40	wet	400ppm
3	A110	OH^-	25	dry	5000ppm
3	A110	CO_3^{2-}	30	medium	1%
4	FPA54	OH^-	25	wet	5000ppm
4	FPA54	CO_3^{2-}	40	dry	1%
4	FPA54	PO_4^{3-}	30	medium	400ppm
5	IRA910	PO_4^{3-}	60	dry	5000ppm
5	IRA910	OH^-	25	medium	1%
5	IRA910	CO_3^{2-}	40	wet	400ppm
6	IRA458	OH^-	40	dry	1%
6	IRA458	PO_4^{3-}	25	wet	5000ppm
6	IRA458	CO_3^{2-}	60	medium	400ppm

Table S6. Comparison of the final model with naive baselines on the test set, evaluated on the same 10-seed grouped train/test splits (mean standard deviation across seeds). The naive baselines are reference predictors that use no learned feature relationships: a global-mean predictor (always predicting the training set mean capacity) and group mean predictors (predicting the mean capacity of the test point's category, stratified by functional group or by anion). They provide a reference against which the learned model's improvement is measured.

Predictor	R^2	RMSE	MAE
LightGBM (final model)	0.702 ± 0.108	0.164 ± 0.040	0.118 ± 0.028
Global mean	-0.032 ± 0.043	0.309 ± 0.038	0.239 ± 0.021
Group mean (FG)	0.292 ± 0.158	0.253 ± 0.033	0.189 ± 0.020
Group mean (anion pKa)	0.105 ± 0.072	0.287 ± 0.030	0.228 ± 0.019

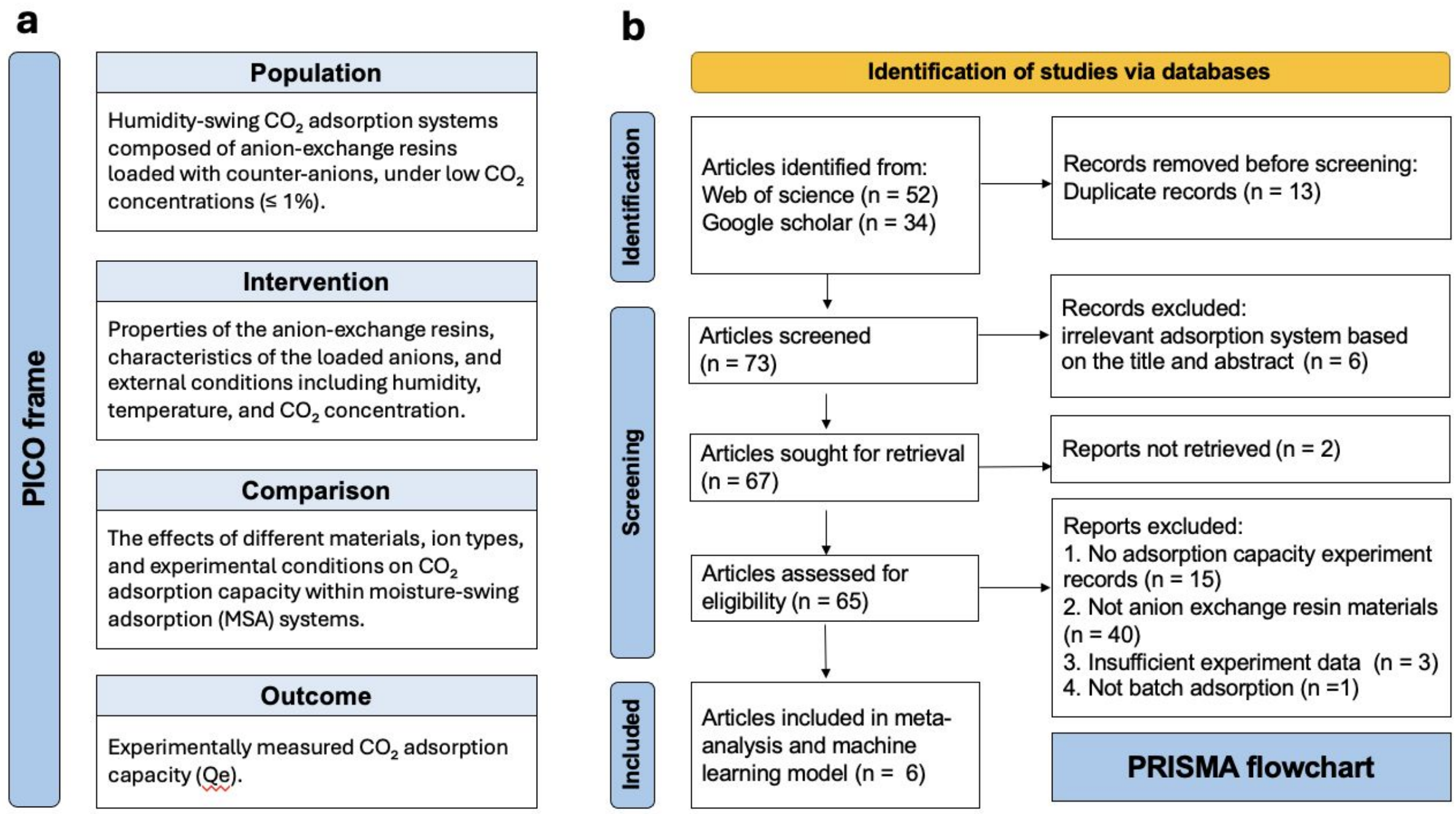

Figure S1. Screening process for articles included in the dataset. (a) PICO framework, and (b) PRISMA flowchart.

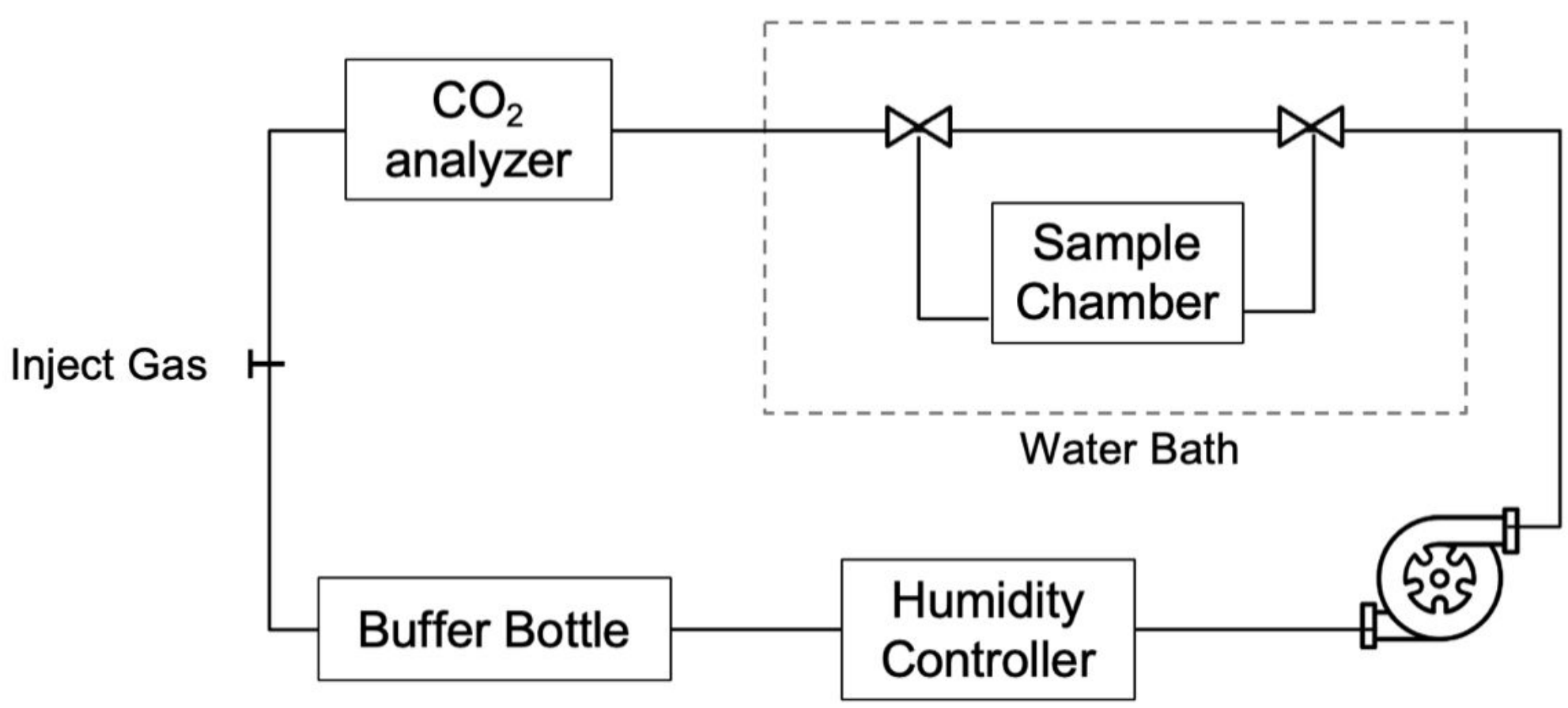

Figure S2. Adsorption experimental system setup.

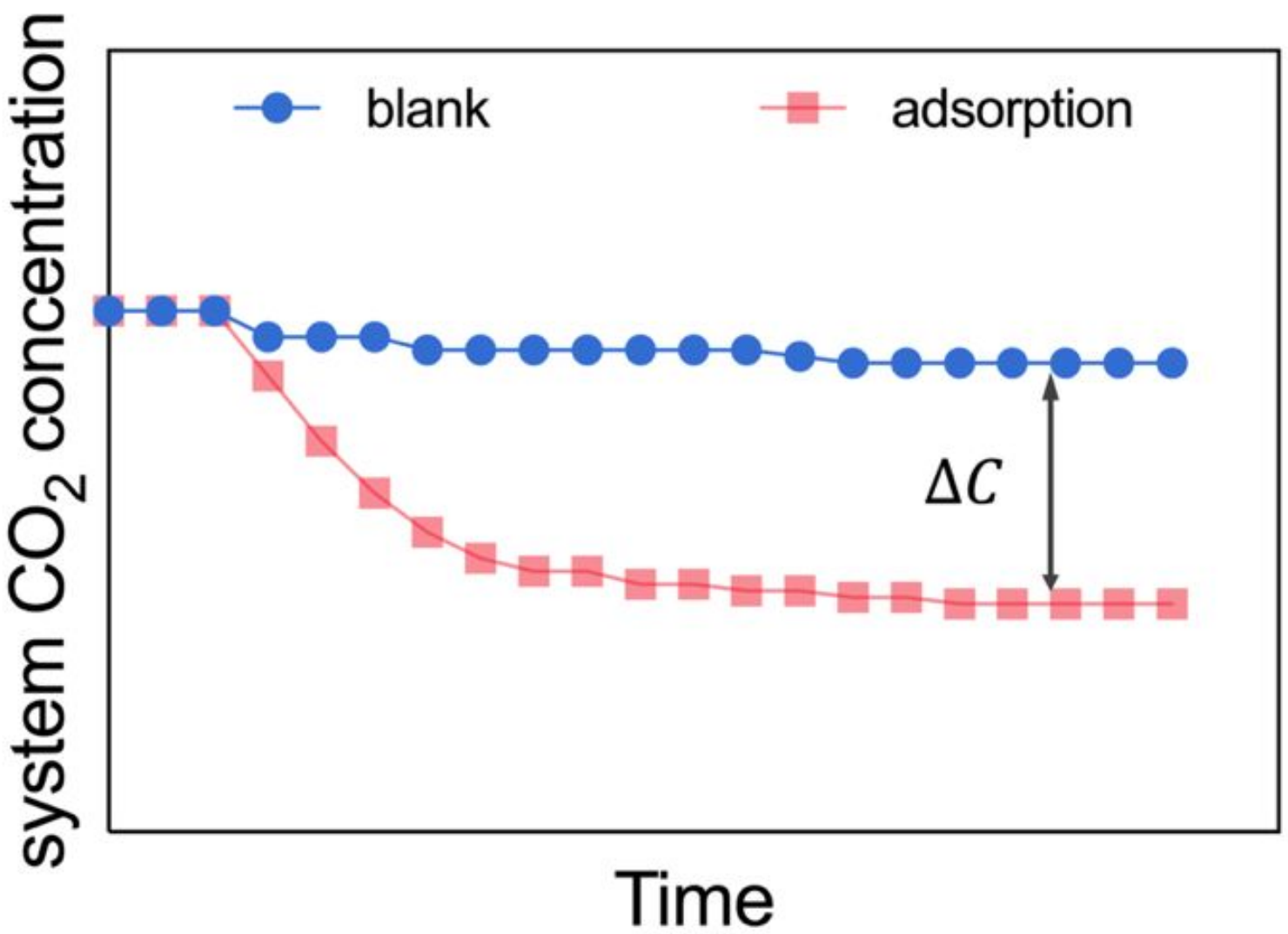

Figure S3. Example CO_2 concentration profiles during adsorption experiments. Blue curve: blank test conducted without resin to account for the contribution of these factors; red curve: adsorption test with resin sample. The equilibrium adsorption capacity was calculated from the concentration difference (ΔC) between blank and sample systems, normalized by resin mass and system volume.

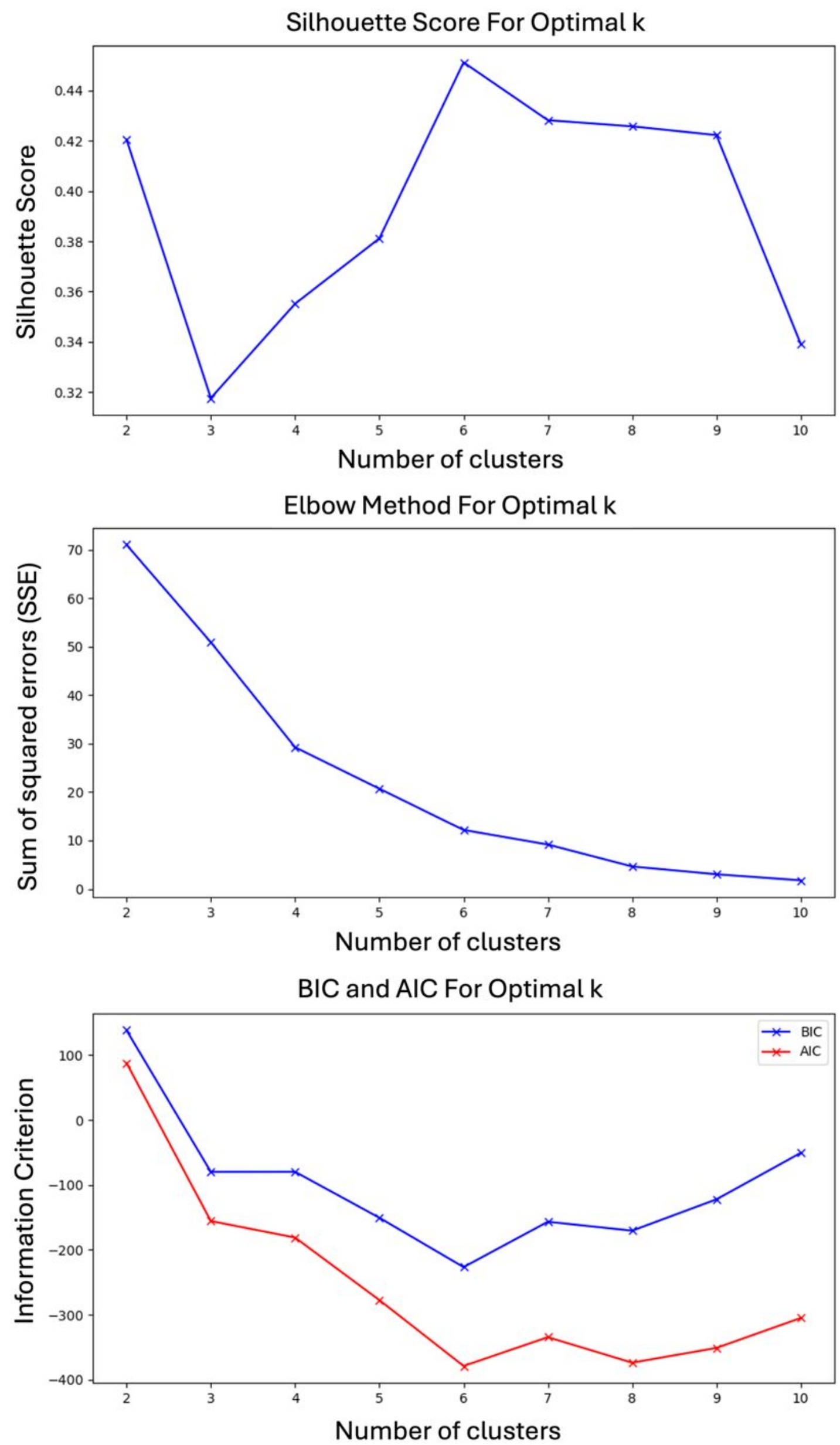

Figure S4. Determination of the optimal number of clusters (K) for resin grouping, assessed by multiple methods. (a) Silhouette scores for K = 2–10, highest at K = 6. (b) Elbow method (within-cluster sum of squared errors, SSE), showing a pronounced elbow at K = 6. (c) Bayesian and Akaike information criteria (BIC and AIC), both minimized at K = 6. All methods consistently support K = 6, which was therefore grouped into 6 clusters.

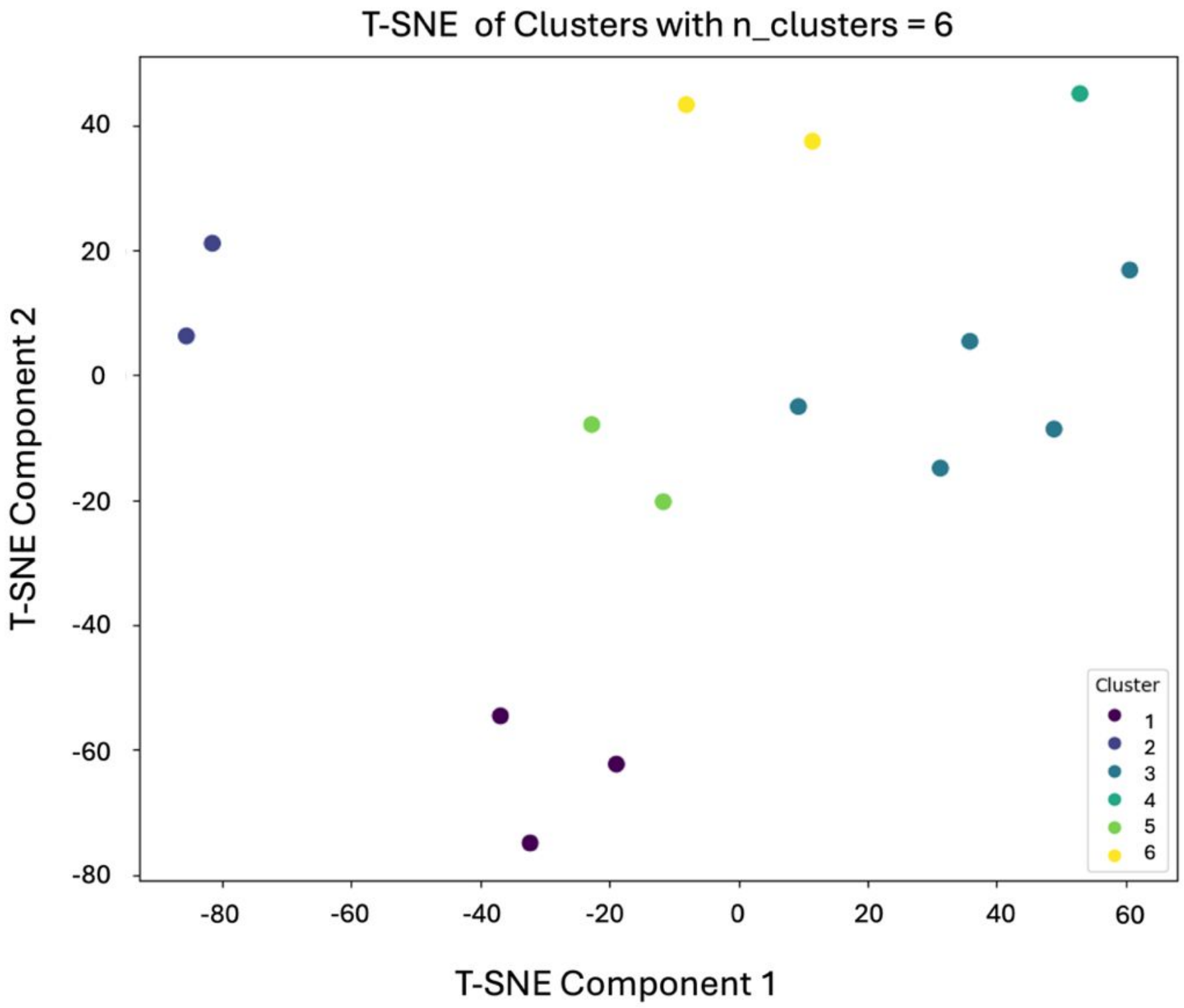

Figure S5. t-distributed stochastic neighbor embedding (T-SNE) visualization of resin clustering based on physicochemical properties of the resins.

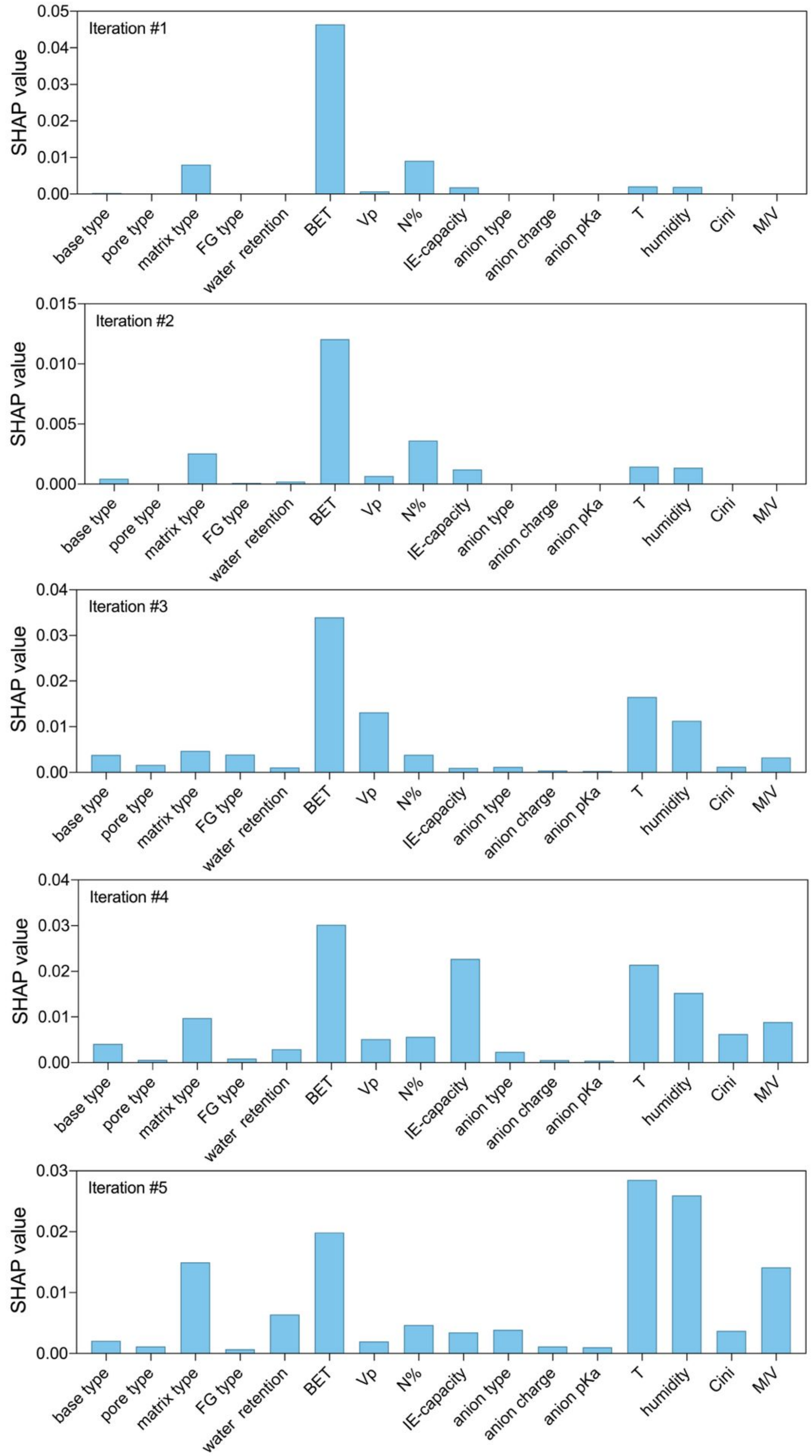

Figure S6. SHAP feature contributions to prediction uncertainty across AL iterations.

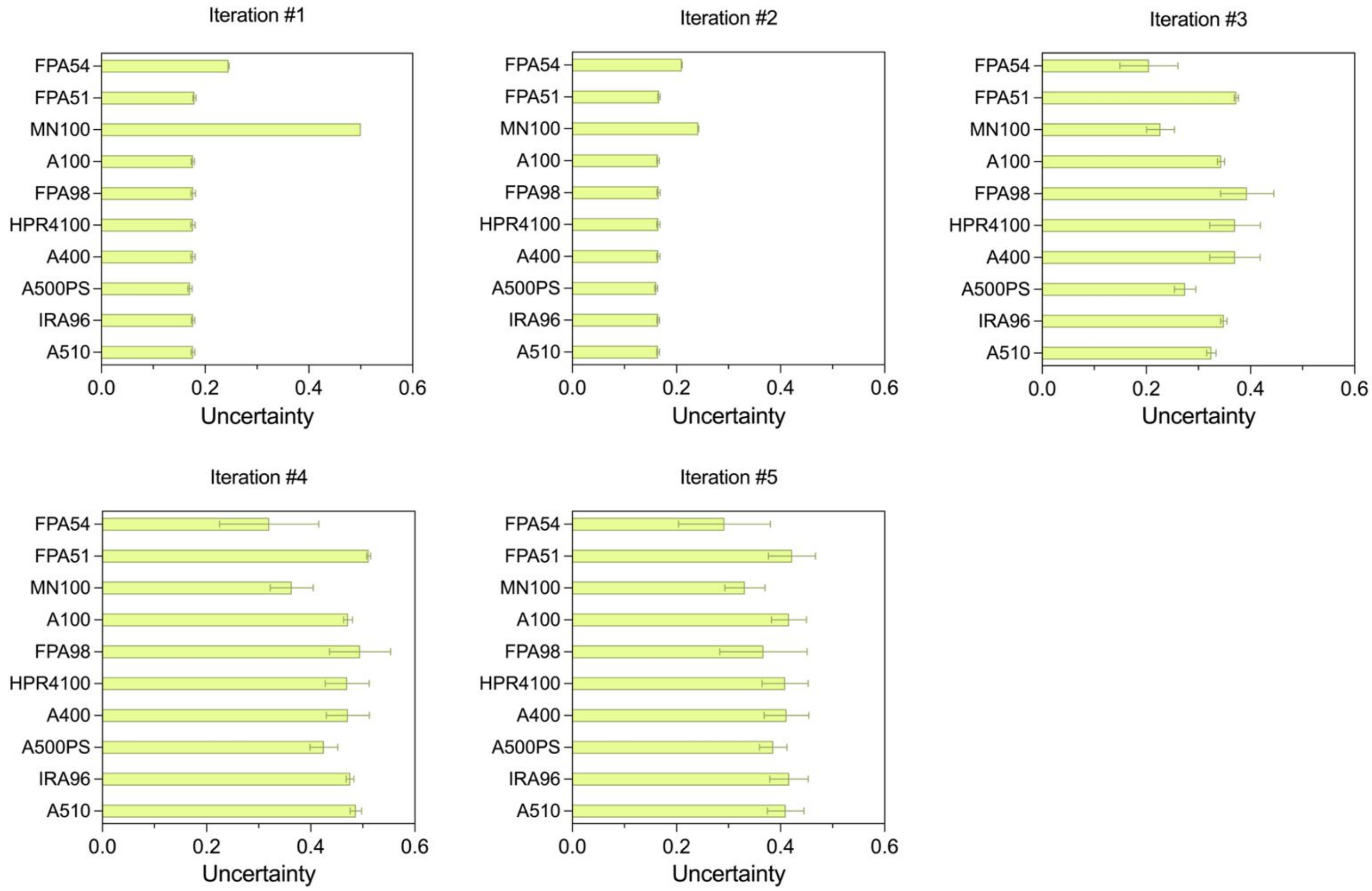

Figure S7. Evolution of resin-specific prediction uncertainty across five AL iterations.

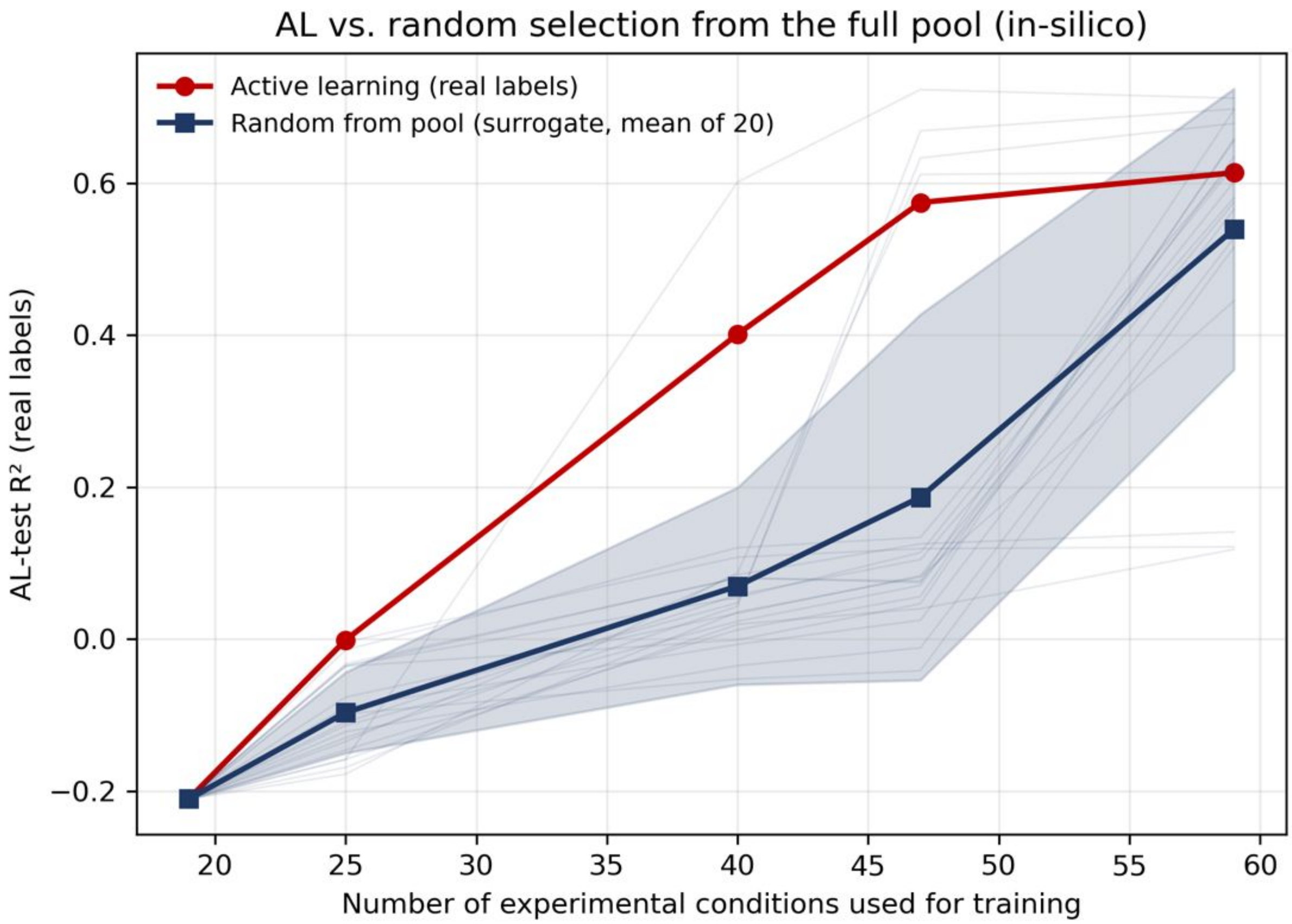

Figure S8. Retrospective in-silico comparison of active learning against random selection from the full candidate pool. The x-axis is the number of experimental conditions used for training; the y-axis is the R^2 on the AL-test set, evaluated with real measured labels. The red curve is the actual active-learning. The blue curve is the mean of 20 random-selection runs that used the same batch sizes and the same AL-test set. The shaded blue band spans the standard deviation across the 20 random runs, and the faint grey lines show the 20 individual random trajectories. At matched training sizes the active-learning trajectory attains higher AL-test R^2 than random selection across the trajectory, most clearly at intermediate sizes. This comparison is an in-silico reference indicative of the value of AL sampling rather than an unbiased benchmark.

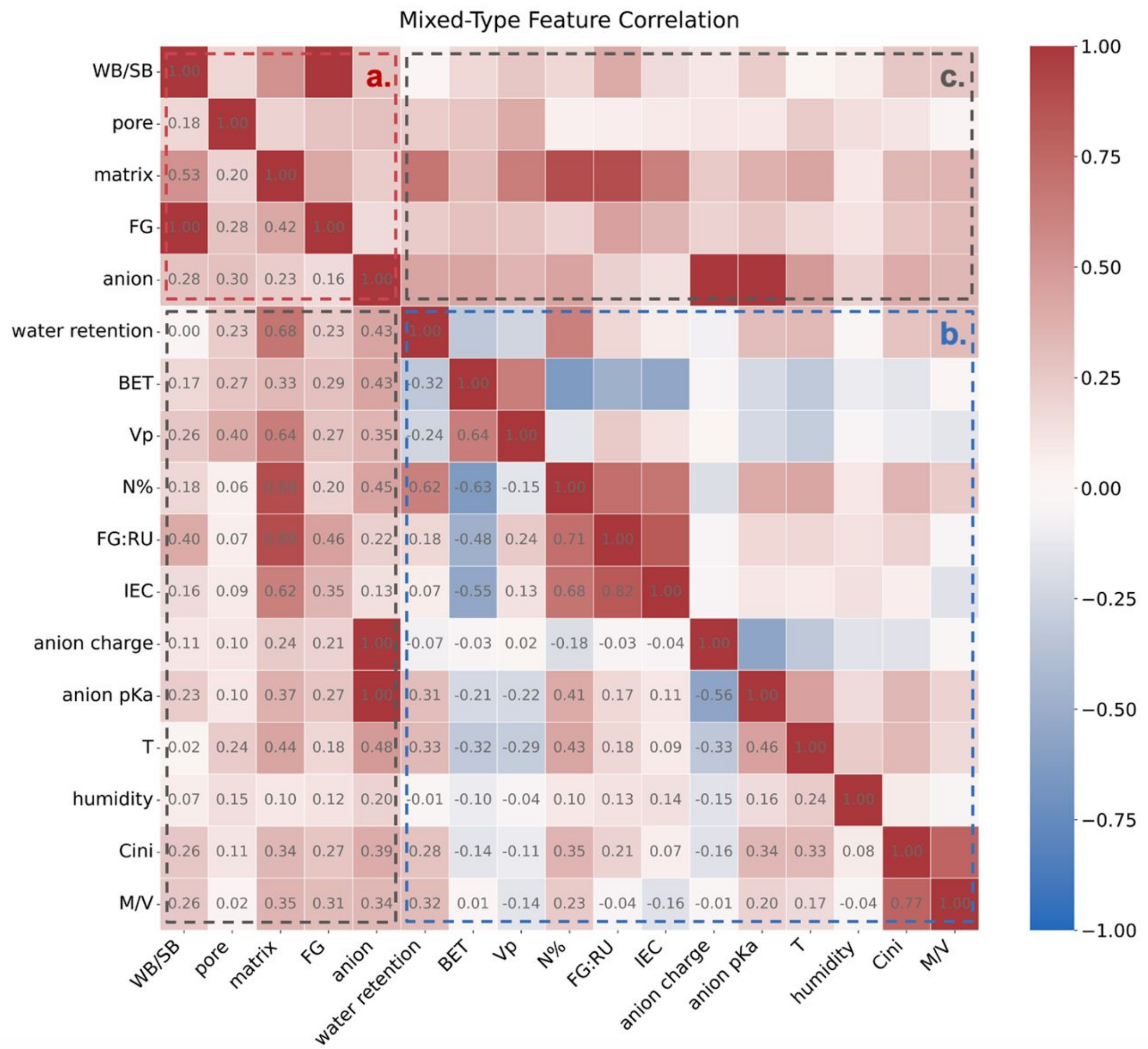

Figure S9. Correlation matrix heatmap of all input features. (a) Categorical–categorical feature correlations (Cramér's V). (b) Numerical–numerical feature correlations (Spearman's ρ). (c) Categorical–numerical feature correlations (correlation ratio). Color scale ranges from blue (−1) to red (+1). Gray values in each cell indicate the corresponding correlation coefficient.

Based on this correlation analysis, the final feature set selected for subsequent modeling included: pore, matrix, Functional group type (FG), water retention, BET, pore volume (Vp), N%, FG:RU, ion exchange capacity (IEC), anion charge, anion pKa, temperature (T), humidity, initial CO_2 concentration (Cini), mass to volume ratio (M/V). Note that counter-anion identity, considered in the AL and meta-analysis, was represented in the final model by two physical descriptors (anion charge and anion pKa) rather than as a categorical variable, to enable generalization to anions characterized by these properties.

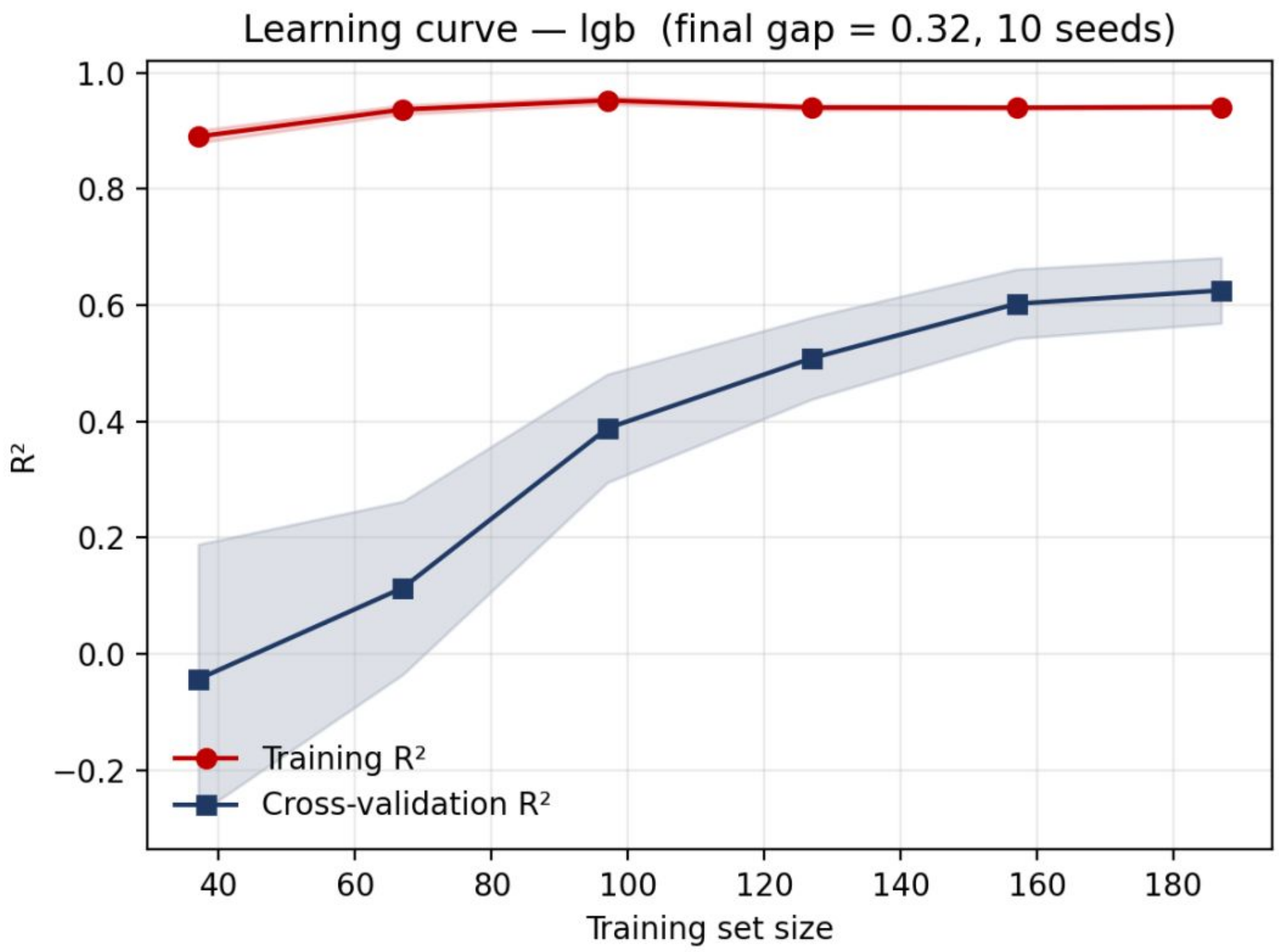

Figure S10. Learning curve of the final model (LightGBM), indicating a data-limited regime. Cross-validated test R^2 (mean ± std over 10 seeds) is shown as a function of training-set size, rising from near zero to 0.62, and has not saturated, although the rate of improvement slows at the largest sizes. This continued improvement with additional data indicates that the train-test gap arises substantially from the limited dataset size, although some degree of overfitting cannot be fully excluded.

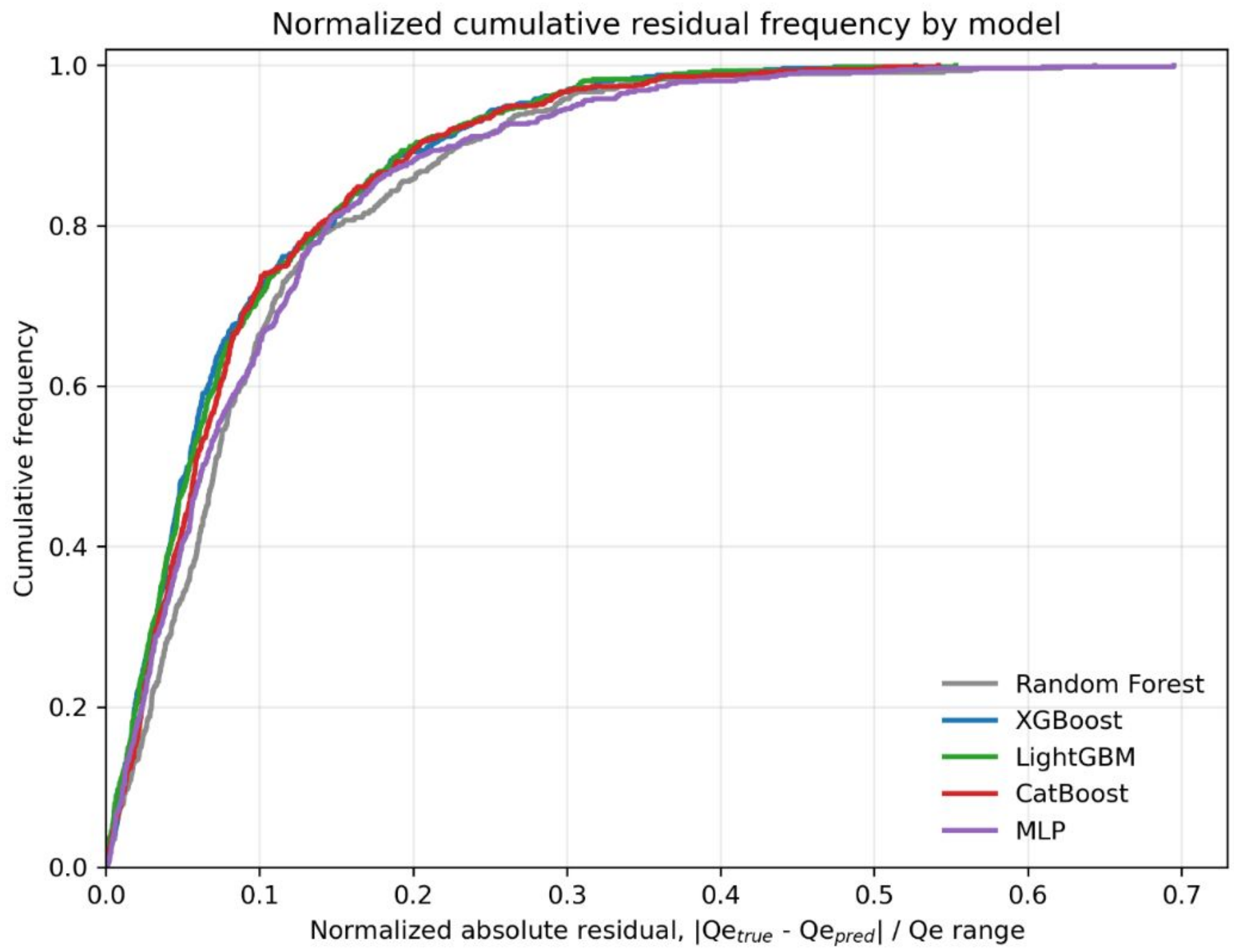

Figure S11. Normalized cumulative residual frequency curves for the five ML models. The normalized residual was calculated as $|Q_{e,pred} - Q_{e,exp}|/(Q_{e,max} - Q_{e,min})$, where $Q_{e,max}$ and $Q_{e,min}$ represent the maximum and minimum experimental Qe values in the test predictions. The y-axis denotes the cumulative fraction of test samples with normalized residuals below a given threshold. A steeper and more left-shifted curve indicates that a larger proportion of predictions fall within a smaller error range, reflecting higher predictive reliability. Among the models, LightGBM showed the highest cumulative fractions at normalized residual thresholds of 0.15 and 0.20, further supporting its selection for subsequent model interpretation.

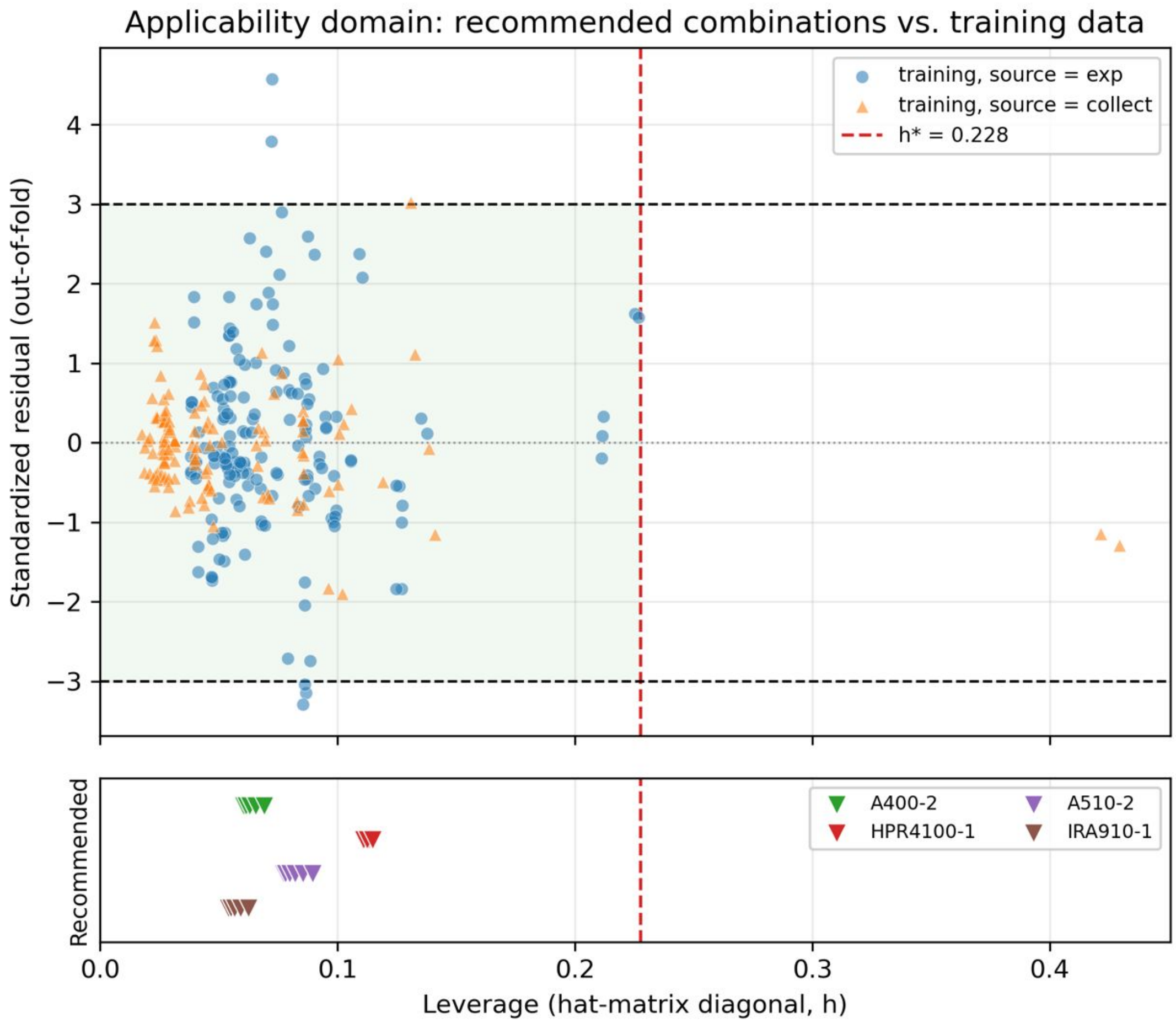

Figure S12. Applicability-domain analysis of the final LightGBM model. (a) The Williams plot based on out-of-fold predictions. Standardized residuals were plotted against leverage values (h, the diagonal of the hat matrix computed in the model's preprocessed feature space). The vertical dashed line marks the warning leverage $h^* = 3(p+1)/n = 0.228$, where p is the number of preprocessed features and n = 290 the number of data points; the horizontal dashed lines mark ±3 standardized residuals. Points falling inside the shaded region ($h < h^*$ and |standardized residual| < 3) lie within the applicability domain (282 of 290, 97.2%). The two high-leverage samples corresponded to IRA67RF, a gel-type resin with an uncommon polyacrylic-matrix/tertiary-amine combination. The few response outliers were also gel-type resins under extreme environmental conditions, such as A420S at high temperature under humid or dry conditions, likely reflecting

the nonlinear and sparsely sampled humidity-dependent behavior of this resin class. (b) Leverage of the recommended resin-anion combinations over the studied humidity range, plotted on the same leverage axis; each marker corresponds to one humidity level. These combinations are characterized by leverage: markers to the left of h^* indicate interpolation, whereas markers beyond h^* indicate extrapolation. All recommended conditions fall within the applicability domain.

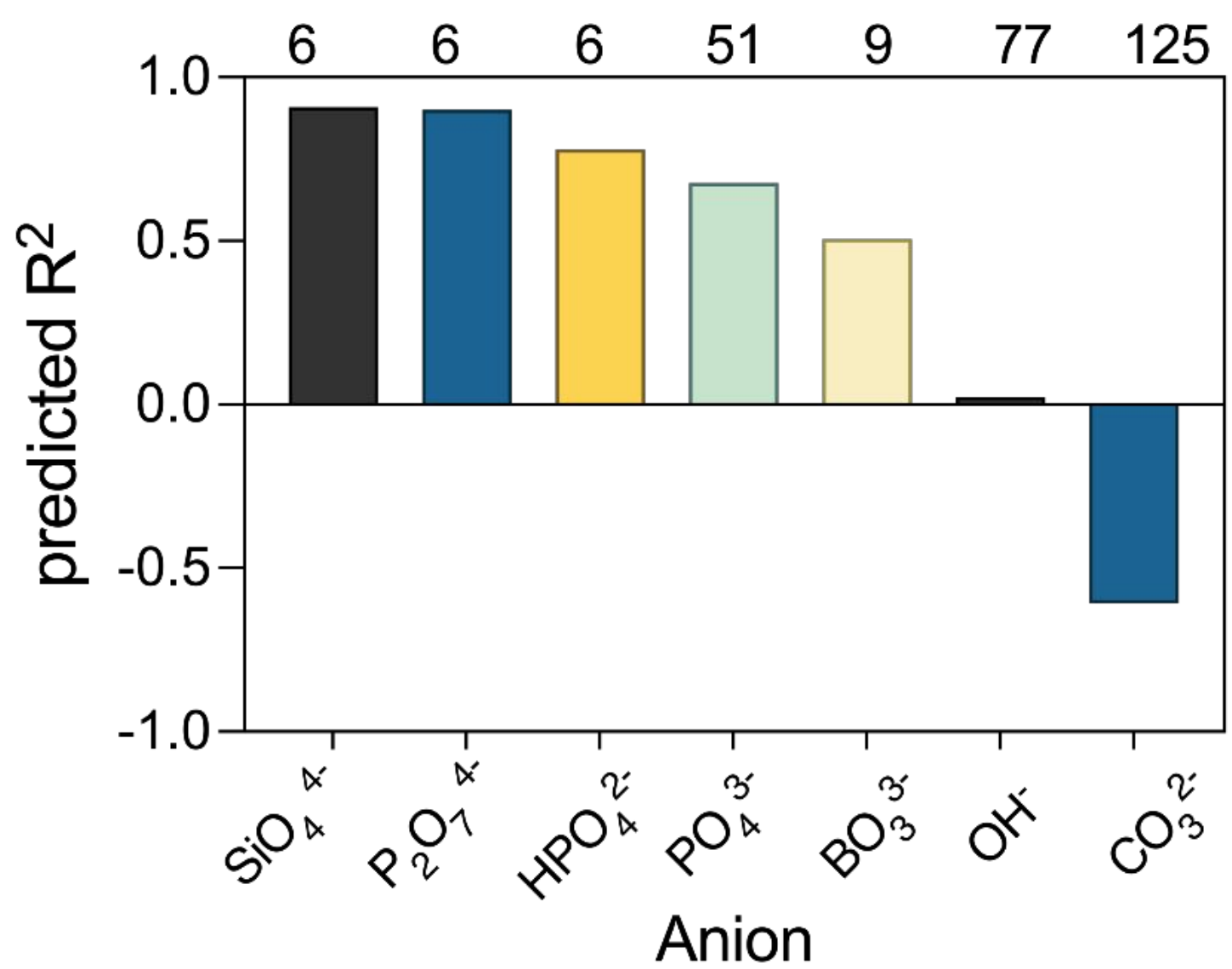

Figure S13. Predictive performance of the model on each anion type during leave-one-anion-out cross-validation. Numbers above each bar show the number of data points for each anion when it served as the test set.

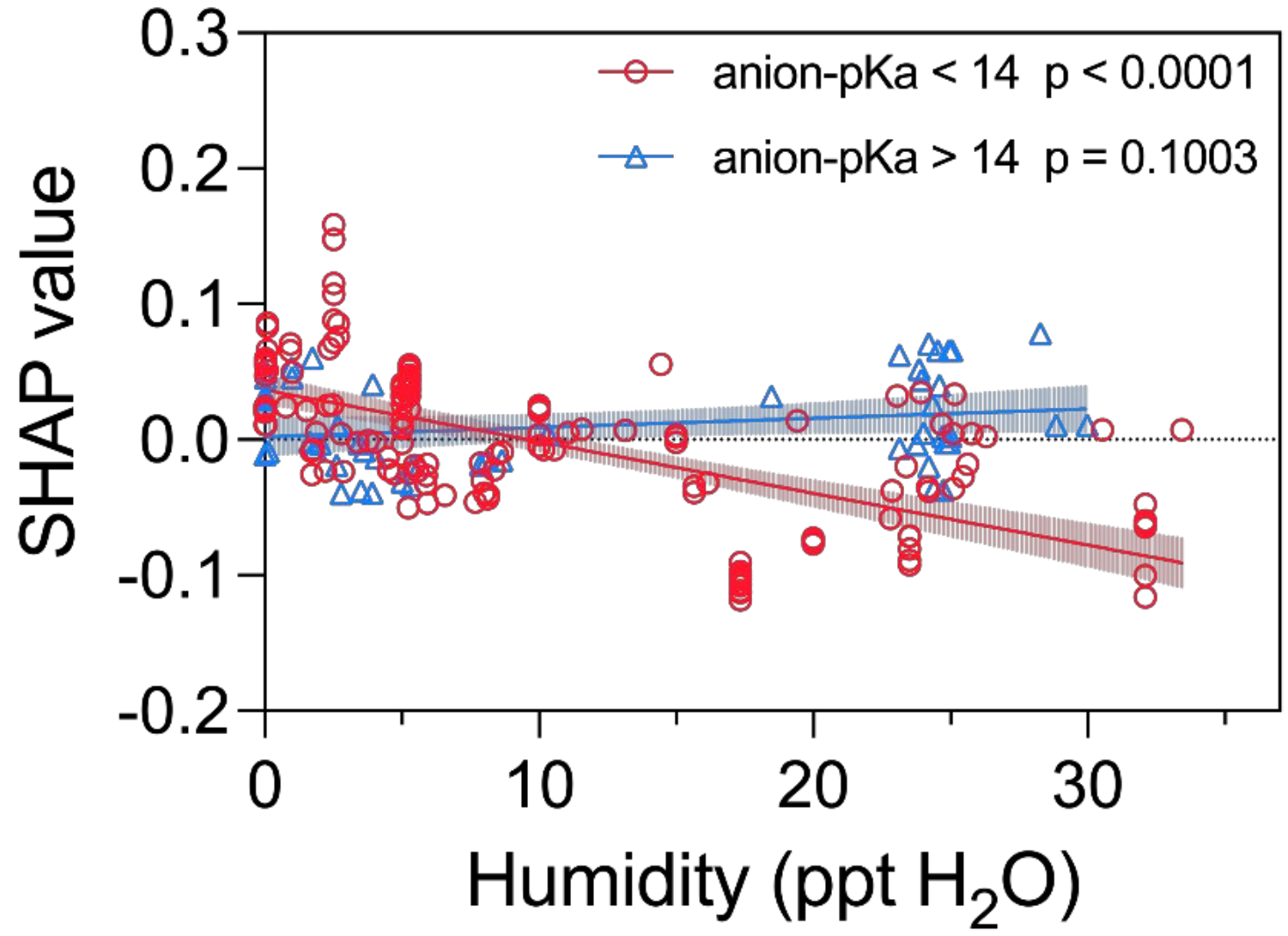

Figure S14. SHAP dependence plot for the humidity feature. Red points represent resins loaded with strong-base anions, while blue points correspond to weak-base anions. The solid curve indicates the fitted trend, with the shaded area representing the 95% confidence interval.

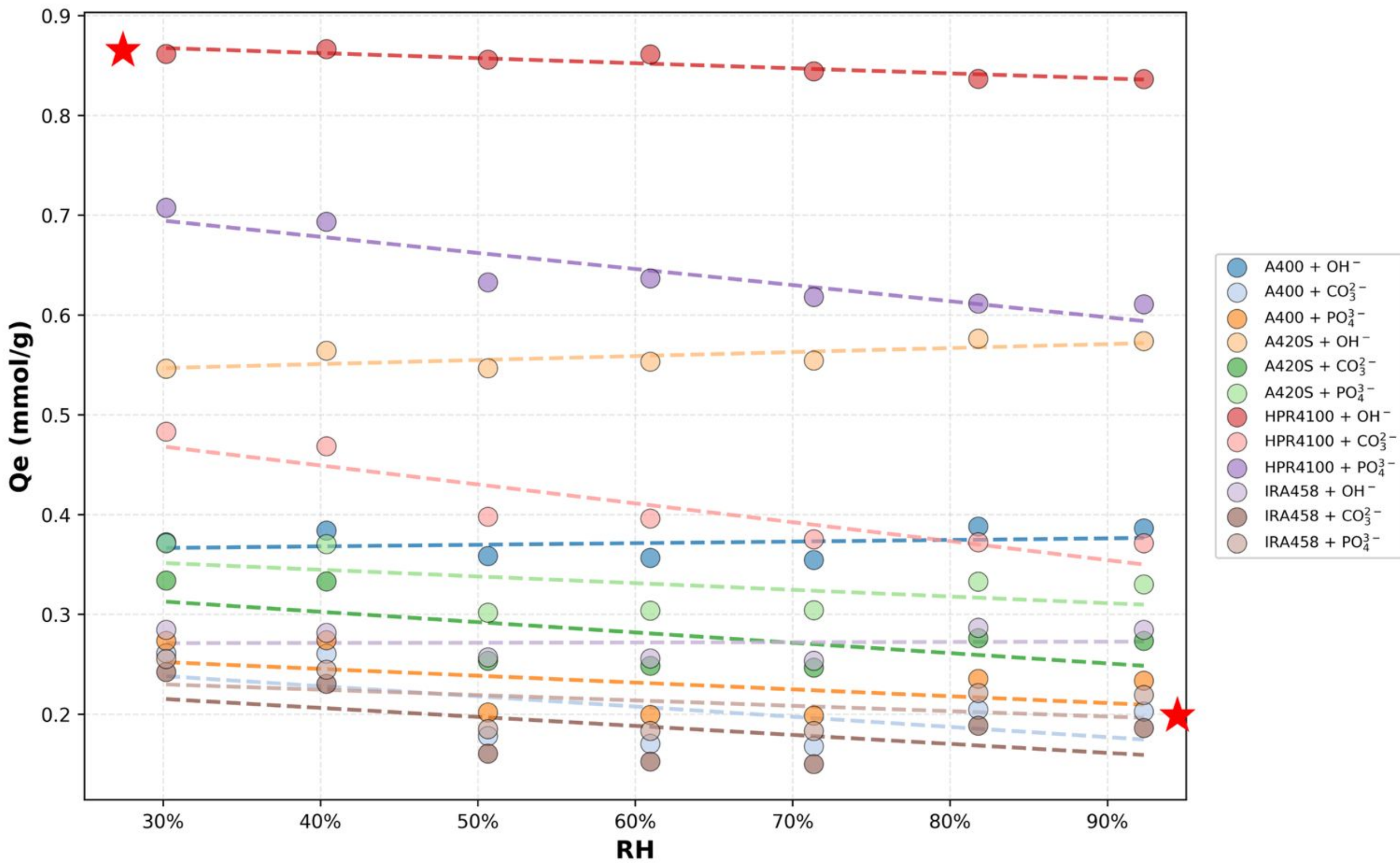

Figure S15. Predicted CO_2 adsorption capacity for gel-type resins as a function of relative humidity (30–90% RH). Stars (★) denote the resin-anion combinations selected for subsequent experimental validation.

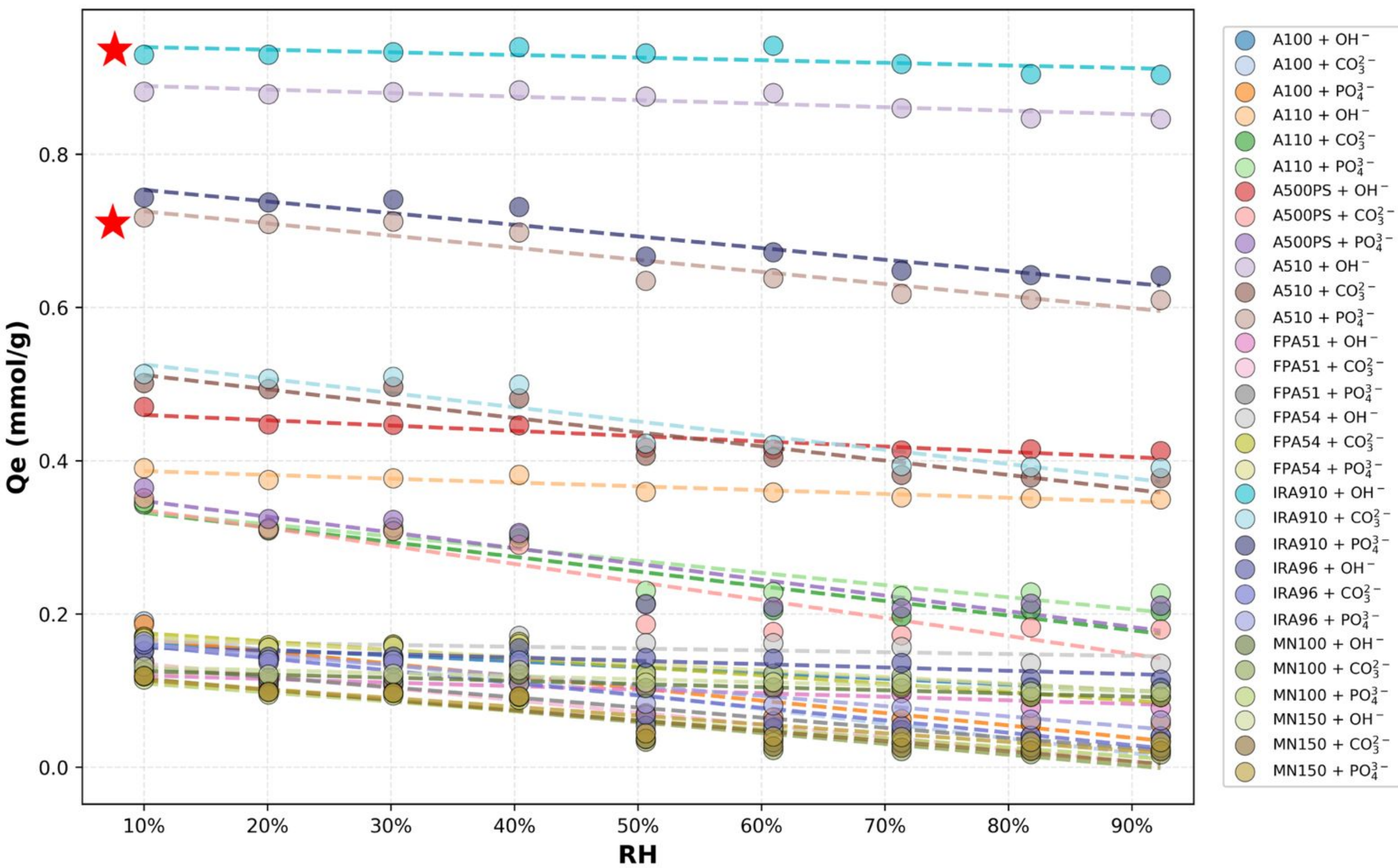

Figure S16. Predicted CO_2 adsorption capacity for macroporous-type resins as a function of relative humidity (10–90% RH). Stars (★) denote the resin-anion combinations selected for subsequent experimental validation.

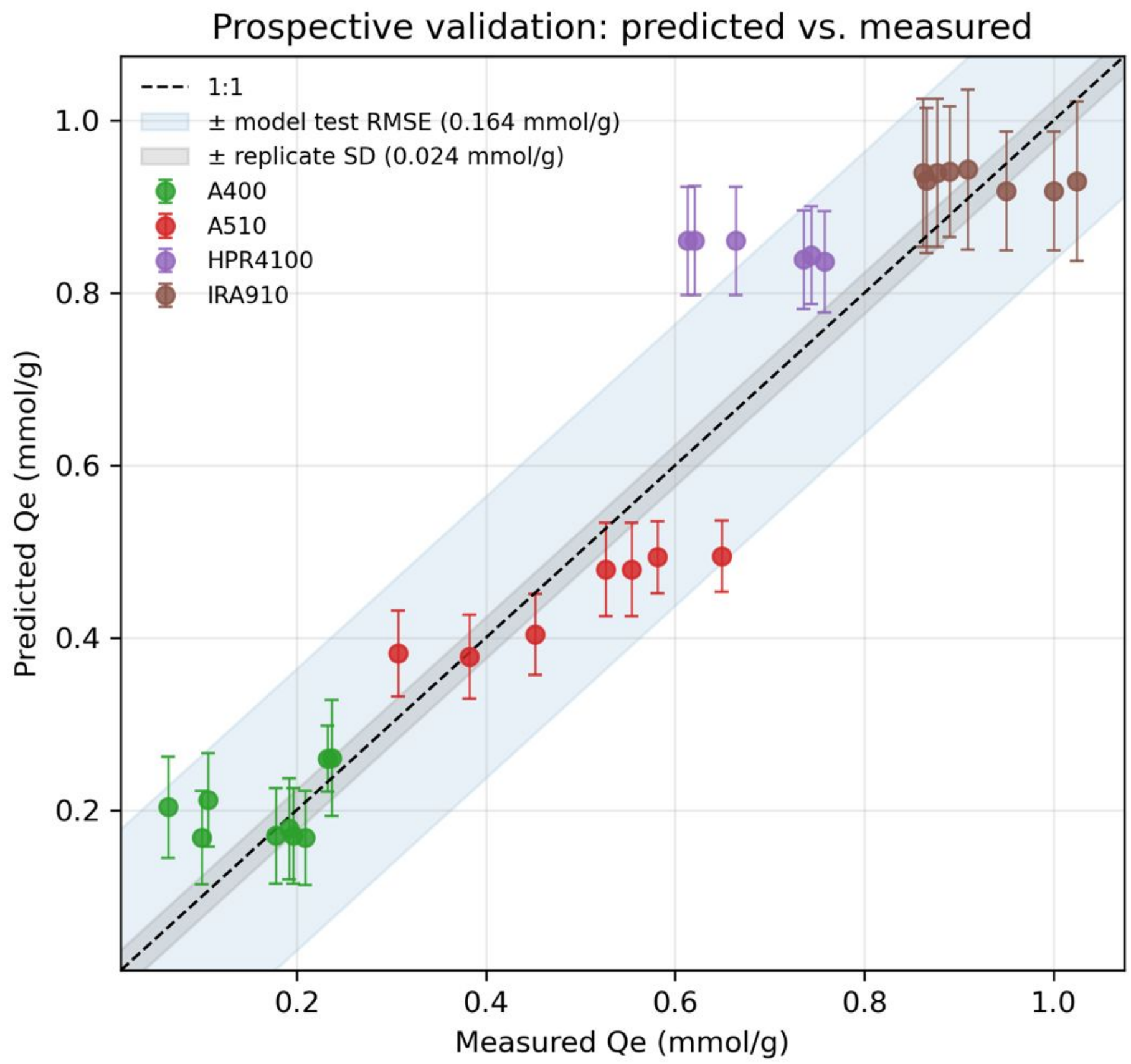

Figure S17. Prospective validation parity plot comparing model-predicted and experimentally measured Qe values for the recommended resin-anion-humidity combinations. Each point represents an individual experimental condition, colored by resin type, with vertical error bars indicating the prediction variability across the 10 random state models. The dashed black line denotes the ideal 1:1 agreement between prediction and experiment. The blue shaded band represents the final model test RMSE (0.164 mmol/g), and the gray shaded band represents the average experimental replicate standard deviation (0.024 mmol/g). Most validation points fall within or close to the model-RMSE band, indicating that the prospective measurements are generally consistent with the model predictions, while the larger deviations observed for HPR4100/OH^- highlight increased uncertainty for less-represented resin-anion chemistries.

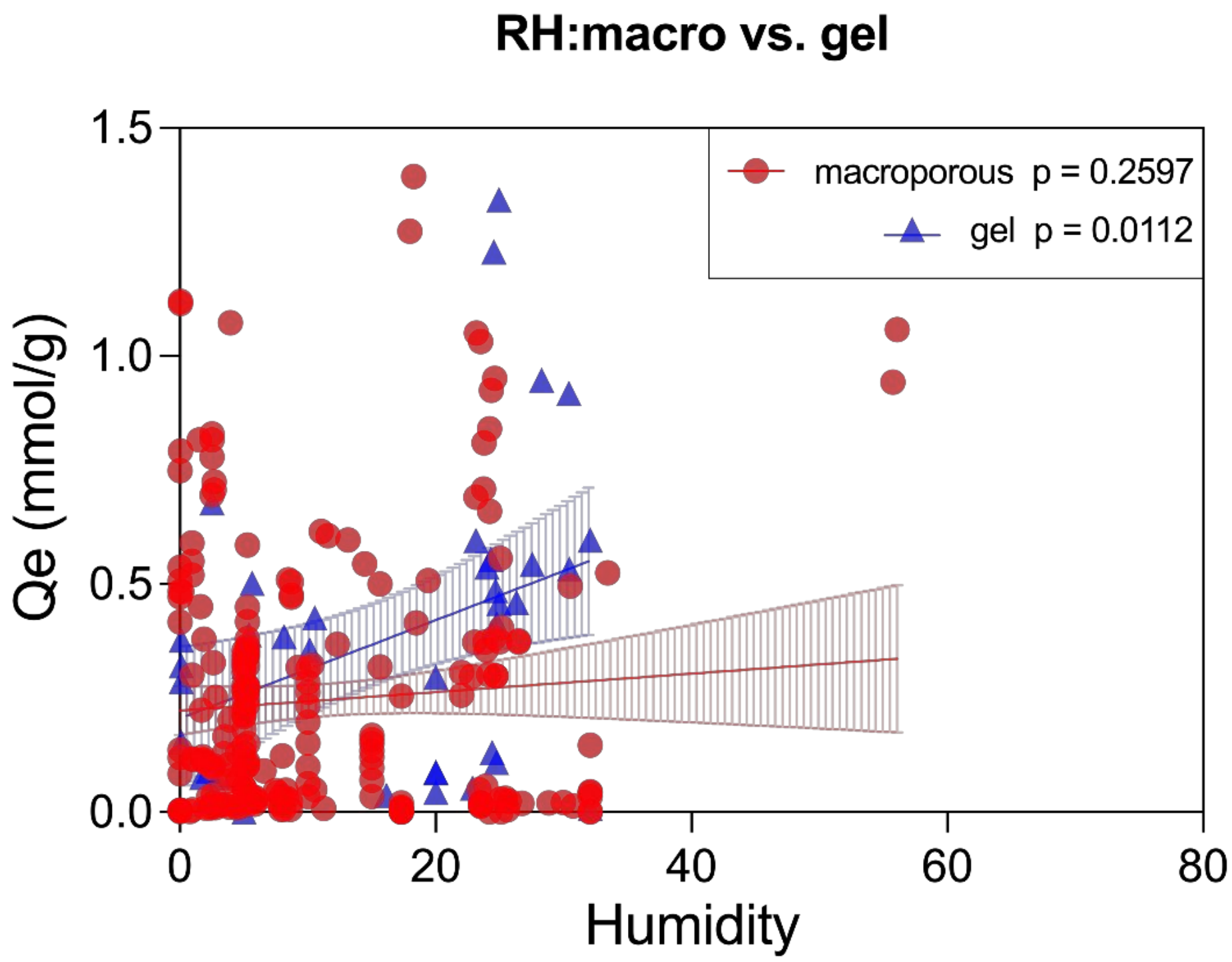

Figure S18. Meta-analysis of humidity effects on CO_2 adsorption capacity, stratified by resin matrix type. Red circles represent all macroporous resins; blue triangles represent all gel-type resins. Trend lines show linear fits for each category, with shaded regions indicating 95% confidence intervals.

References

(1) Wang, X.; Song, J.; Chen, Y.; Xiao, H.; Shi, X.; Liu, Y.; Zhu, L.; He, Y.-L.; Chen, X. CO2 absorption over ion exchange resins: the effect of amine functional groups and microporous structures. *Industrial & engineering chemistry research* **2020**, *59* (38), 16507–16515.

(2) Song, J.; Zhu, L.; Shi, X.; Liu, Y.; Xiao, H.; Chen, X. Moisture swing ion-exchange resin-PO4 sorbent for reversible CO2 capture from ambient air. *Energy & fuels* **2019**, *33* (7), 6562–6567.

(3) Hegarty, J.; Shindel, B.; Sukhareva, D.; Barsoum, M. L.; Farha, O. K.; Dravid, V. Expanding the Library of Ions for Moisture-Swing Carbon Capture. *Environmental Science & Technology* **2023**, *57* (50), 21080–21091.

(4) Zhu, Y.; Booth, A.; Hatzell, K. B. Confinement Effects on Moisture-Swing Direct Air Capture. *Environmental Science & Technology Letters* **2024**, *11* (2), 89–94.

(5) Wang, T.; Wang, X.; Hou, C.; Liu, J. Quaternary functionalized mesoporous adsorbents for ultra-high kinetics of CO2 capture from air. *Scientific reports* **2020**, *10* (1), 21429.

(6) Wang, T.; Lackner, K. S.; Wright, A. Moisture swing sorbent for carbon dioxide capture from ambient air. *Environmental science & technology* **2011**, *45* (15), 6670–6675.

(7) Yuan, X.; Suvarna, M.; Lim, J. Y.; Pérez-Ramírez, J.; Wang, X.; Ok, Y. S. Active learning-based guided synthesis of engineered biochar for CO2 capture. *Environmental Science & Technology* **2024**, *58* (15), 6628–6636.

(8) Zhong, S.; Lambeth, D. R.; Igou, T. K.; Chen, Y. Enlarging applicability domain of quantitative structure–activity relationship models through uncertainty-based active learning. *ACS ES&T Engineering* **2022**, *2* (7), 1211–1220.
